# The presubiculum is preserved from neurodegenerative changes in Alzheimer’s disease

**DOI:** 10.1186/s40478-018-0563-8

**Published:** 2018-07-20

**Authors:** Christina E. Murray, Priya Gami-Patel, Eleni Gkanatsiou, Gunnar Brinkmalm, Erik Portelius, Oliver Wirths, Wendy Heywood, Kaj Blennow, Jorge Ghiso, Janice L. Holton, Kevin Mills, Henrik Zetterberg, Tamas Revesz, Tammaryn Lashley

**Affiliations:** 1 The Queen Square Brain Bank for Neurological Disorders, Department of Molecular Neuroscience, UCL Institute of Neurology, London, WC1N 3BG UK; 2 Institute of Neuroscience and Physiology, Department of Psychiatry and Neurochemistry, The Sahlgrenska Academy at the University of Gothenburg, Mölndal, Sweden; 3 Clinical Neurochemistry Laboratory, Sahlgrenska University Hospital, Mölndal, Sweden; 4 Department of Psychiatry and Psychotherapy, University Medical Center (UMG), Georg-August-University, Goettingen, Germany; 5 Centre for Translational Omics, Great Ormond Street Institute of Child health, UCL, London, UK; 6 New York University, New York, NY USA; 7 Dementia Research Institute, UCL , London, UK

**Keywords:** Alzheimer’s disease, Presubiculum, Amyloid, Tau, Neuroinflammation

## Abstract

In the majority of affected brain regions the pathological hallmarks of Alzheimer’s disease (AD) are β-amyloid (Aβ) deposits in the form of diffuse and neuritic plaques, tau pathology in the form of neurofibrillary tangles, neuropil threads and plaque-associated abnormal neurites in combination with an inflammatory response. However, the anatomical area of the presubiculum, is characterised by the presence of a single large evenly distributed ‘lake-like’ Aβ deposit with minimal tau deposition or accumulation of inflammatory markers. Post-mortem brain samples from sporadic AD (SAD) and familial AD (FAD) and two hereditary cerebral amyloid diseases, familial British dementia (FBD) and familial Danish dementia (FDD) were used to compare the morphology of the extracellular proteins deposited in the presubiculum compared to the entorhinal cortex. The level of tau pathology and the extent of microglial activation were quantitated in the two brain regions in SAD and FAD. Frozen tissue was used to investigate the Aβ species and proteomic differences between the two regions. Consistent with our previous investigations of FBD and FDD cases we were able to establish that the ‘lake-like’ pre-amyloid deposits of the presubiculum were not a unique feature of AD but they also found two non-Aβ amyloidosis. Comparing the presubiculum to the entorhinal cortex the number of neurofibrillary tangles and tau load were significantly reduced; there was a reduction in microglial activation; there were differences in the Aβ profiles and the investigation of the whole proteome showed significant changes in different protein pathways. In summary, understanding why the presubiculum has a different morphological appearance, biochemical and proteomic makeup compared to surrounding brain regions severely affected by neurodegeneration could lead us to understanding protective mechanisms in neurodegenerative diseases.

**Electronic supplementary material:**

The online version of this article (10.1186/s40478-018-0563-8) contains supplementary material, which is available to authorized users.

## Introduction

The hippocampus and surrounding parahippocampal region are important for memory function and are severely affected early in Alzheimer’s disease (AD) [2, 84]. The pathology observed in the hippocampus consists of marked neuronal loss, severe β-amyloid (Aβ) plaque deposition, neurofibrillary tangle (NFT) formation and a neuroinflammatory reaction. However, one area within the parahippocampus, the presubiculum, appears to have a different pathological profile in comparison to other areas of the medial temporal region [2, 36, 37, 84]. In AD, diffuse ‘lake-like’ Aβ deposits appear in the presubiculum early in the disease process corresponding to Thal phases 2 and 3 with the morphology of the deposits not changing through to end stage disease, Thal stage 5 [74].

The hippocampus proper is formed from the dentate gyrus, the CA3/4, CA2, CA1 areas and the subiculum. The adjacent parahippocampal region is connected to the hippocampus via the perforant pathway [4, 10, 79]. The parahippocampus is made up of the pre- and parasubiculum, transsubiculum and entorhinal cortex [17]. It has been shown that the presubiculum has different pathological properties to the hippocampal formation in AD [2, 37, 84], in the form that the presubiculum contained a ‘lake-like amyloid deposit’ composed of Aβ that filled the anatomical area instead of forming defined Aβ plaques [37]. Thioflavin S and Congo-red staining, which highlight misfolded protein aggregates in an amyloid β-sheet conformational state, confirmed that the Aβ deposited in the presubiculum was diffuse or pre-amyloid in nature [2, 75, 84]. A further number of observational differences were seen between the presubiculum and neighbouring hippocampal areas in that, only sparse NFTs, no activated glial cells and no amyloid-associated proteins such as apolipoprotein E or apolipoprotein J (clusterin) were detected [2, 36, 37, 84]. Conversely, the neighbouring entorhinal cortex had severe neuronal loss, dense core amyloid plaques, frequent NFT’s, activated microglia and activated astrocytes [2, 36, 37, 84]. These previous studies used silver impregnation techniques to identify the amyloid and NFTs together with semi-quantitative methods of analysis.

The amyloid plaques present in AD are composed of aggregated Aβ peptides. Aβ is formed from cleavage of the amyloid precursor protein (APP) and a number of different Aβ peptide species are released due to secretases cleaving at different sites [11, 64, 71]. Whereas in the two hereditary neurodegenerative diseases, familial British dementia (FBD) and familial Danish dementia (FDD), both are characterised by extra-cellular amyloid and pre-amyloid deposits. In FBD a point mutation, while in FDD a decamer duplication insertion, abolishes the stop codon of the *BRI2* gene [23, 30, 31, 77, 80, 81] resulting in elongated precursor proteins, from which the amyloid peptides amyloid-Bri (ABri) and amyloid-Dan (ADan) are released in FBD and FDD, respectively. Similar to AD, there are numerous ABri and ADan amyloid plaques as well as diffuse plaques and also severe tau pathology in medial temporal lobe structures in both diseases, allowing for comparison with AD.

In this study we investigated whether the unique morphological appearance of deposited Aβ in the presubiculum was protein-specific or due to different tissue factors found in the two different brain regions. To achieve these aims we investigated the morphological appearance of three amyloid forming extracellular peptides found in the presubiculum in SAD, FAD, FBD and FDD using immunohistochemical methods. To determine whether the presubiculum is protected against neurodegeneration and neuroinflammation, in SAD and FAD we quantitated the level of total tau pathology, the number of neurofibrillary tangles and levels of microglial activation compared to the neighbouring entorhinal cortex using immunohistochemical methods. We used laser capture microdissection paired with mass spectrometry to determine the Aβ peptide species that form the extracellular parenchymal deposits in the presubiculum compared to the amyloid plaques found in the entorhinal cortex in SAD and FAD. Laser capture microdissection teamed with label-free proteomics were used to identify altered protein expression or pathways that could be responsible for the differences between the presubiculum and entorhinal cortex.

## Material and methods

### Cases

All sporadic AD cases (SAD; *n* = 19) and familial AD cases (FAD; *n* = 11 (8 *PS1* and 3 *APP* mutations) were obtained through the brain donation program of the Queen Square Brain Bank for Neurological Disorders (QSBB), Department of Molecular Neuroscience, UCL Institute of Neurology. The FBD (*n* = 4) and FDD (*n* = 4) brains were donated to the QSBB, the Neuropathology Department, Århus Kommunehospital, Århus, Denmark or the Division of Neuropathology Department, UCL Institute of Neurology. The criteria for the neuropathological diagnosis of FBD and FDD have been previously described [30, 31] while for that of AD standard diagnostic criteria were used [9, 52, 74]. The demographic data for all cases are shown in Table 1. Ethical approval for the study was obtained from the Local Research Ethics Committee of the National Hospital for Neurology and Neurosurgery.

**Table 1:** Demographics of cases used in the study

Cases	Disease group	Gene/Mutations	Age at onset	Age at death	Disease duration	Sex	Post-mortem delay (hours)	Fixation time (weeks)	Braak and Braak stage	Thal phase	CERAD score
1	SAD	–	63	73	10	M	31	7.14	6	5	Frequent
2	SAD	–	51	63	12	F	16	4.00	6	5	Frequent
3*	SAD	–	51	62	11	F	63	2.71	6	5	Frequent
4	SAD	–	65	70	5	F	47	4.29	5	5	Moderate
5	SAD	–	64	77	13	M	90	3.14	6	5	Frequent
6	SAD	–	49	62	13	F	77	8.00	6	5	Frequent
7	SAD	–	72	88	16	M	86	3.29	6	5	Frequent
8	SAD	–	52	69	17	M	35	6.14	6	5	Frequent
9	SAD	–	65	72	7	M	39	3.00	5	5	Moderate
10	SAD	–	76	85	9	F	90	3.86	6	5	Frequent
11	SAD	–	55	64	9	M	77	3.43	6	5	Frequent
12	SAD	–	69	74	5	F	94	3.00	6	5	Frequent
13	SAD	–	80	85	5	M	129	n/a	5	5	Moderate
14	SAD	–	46	52	6	F	52	2.57	6	5	Frequent
15	SAD	–	49	54	6	F	48	4.29	6	5	Frequent
16	SAD	–	67	72	5	M	91	4.14	6	5	Frequent
17	SAD	–	65	79	14	F	23	3.43	6	5	Frequent
18	SAD	–	52	68	16	M	35	3.00	6	5	Frequent
19*	SAD	–	58	68	10	M	52	3.86	6	5	Frequent
*SAD summary*			*60.5 (10)*	*70.4 (9.8)*	*9.9 (4.1)*	*10 M:9F*	*61.8 (30)*	*4.1 (1.5)*	*5.8 (0.4)*	*5 (0)*	*Frequent*
20*	FAD	*PS1 Intron 4*	35	52	17	F	33	n/a	6	5	Frequent
21	FAD	*PS1 S132A*	61	70	9	M	n/a	4.29	5	5	Frequent
22	FAD	*PS1 Intron 4*	42	51	9	M	43	4.00	6	5	Frequent
23*	FAD	*APP V717 L*	48	59	11	F	90	n/a	6	5	Frequent
24	FAD	*APP V717I*	60	66	6	M	68	3.14	6	5	Frequent
25	FAD	*PS1 A434T/ T291A*	42	47	5	M	44	4.29	5	5	Frequent
26	FAD	*PS1 R278I*	46	66	20	F	32	4.86	6	5	Frequent
27	FAD	*PS1 I202F*	48	59	11	F	26	4.00	6	5	Frequent
28	FAD	*PS1 E120K*	33	37	4	F	24	6.00	6	5	Frequent
29	FAD	*APP V717I*	44	56	12	F	16	4.29	6	5	Frequent
30	FAD	*PS1 Intron 4*	39	47	8	F	n/a	n/a	6	5	Frequent
*FAD summary*			*45.3 (8.9)*	*55.5 (9.9)*	*10.2 (4.9)*	*4 M:7F*	*53.7 (43.7)*	*4.4 (0.8)*	*5.8 (0.4)*	*5*	*Frequent*
31	FBD	*BRI2*	57	68	11	F	6.5	3.00	5	n/a	n/a
32	FBD	*BRI2*	n/a	56	n/a	F	n/a	n/a	5	n/a	n/a
33	FBD	*BRI2*	n/a	65	n/a	F	n/a	n/a	5	n/a	n/a
34	FBD	*BRI2*	n/a	60	n/a	M	n/a	n/a	5	n/a	n/a
*FBD summary*			*57*	*62.3 (5.3)*	*11*	*1 M:3F*	*6.5*	*3.00*	*5*	*n/a*	*n/a*
36	FDD	*BRI2*	25	43	18	M	n/a	n/a	5	n/a	n/a
37	FDD	*BRI2*	34	60	26	F	n/a	n/a	5	n/a	n/a
38	FDD	*BRI2*	40	58	18	M	n/a	n/a	5	n/a	n/a
39	FDD	*BRI2*	21	53	32	M	n/a	n/a	5	n/a	n/a
*FDD summary*			*30 (8.6)*	*53.5 (7.6)*	*23.5 (6.8)*	*2 M:1F*	*n/a*	*n/a*	*5*	*n/a*	*n/a*

All cases were used for morphological analysis of the presubiculum. ‘*’ cases used for proteomics

### Immunohistochemistry

Eight-micron-thick formalin-fixed paraffin-embedded tissue sections from the hippocampal/parahippocampal region, taken from the level of the lateral geniculate body, were cut from the cases listed in Table 1. Sections were deparaffinised in xylene and rehydrated using graded alcohols. Immunohistochemistry for all antibodies required pre-treatment with a pressure cooker for 10 min in citrate buffer pH 6.0. Aβ, ABri and ADan immunohistochemistry also required formic acid pre-treatment prior to pressure cooking. Endogenous peroxidase activity was blocked in 0.3% H_2_O_2_ in methanol for 10 min and non-specific binding with 10% dried milk solution. Tissue sections were incubated with primary antibodies; Aβ (1:100; Dako), 1–57 N-terminal Aβ_pE3_ (1:1000; Synaptic Systems), 2–48 N-terminal Aβ_pE3_ (1:100; Synaptic Systems), pE-Aβ (1:200; Synaptic Systems), Aβ-pE11 (1:100; Synaptic Systems), Aβ4-x (1:200; [83]); Aβ1–5 (1:500; Synaptic systems 218,231), ABri (338; 1:1000), ADan (5282; 1:1000), AT8 (1:600; Thermo), Iba1 (1:1000; Wako), CD68 (1:100, DAKO), CR3–43 (1:150, DAKO), Annexin A1 (1:4000; Abcam), Annexin 2 (1:2000; Abcam), DOCK2 (1:100; Abcam) and INPPD5 (1:200; Proteintech) for 1 h at RT, followed by biotinylated anti-rabbit IgG (1:200; DAKO) or biotinylated anti-mouse IgG (1:200; DAKO) for 30 min at RT and Avidin-Biotin complex (30 min; Dako). Colour was developed with di-aminobenzidine/H_2_0_2_ [42]. All antibodies were commercially available apart from anti-ABri and anti-ADan which have been previously validated [30, 31]. Stained sections were digitised using a Leica slide scanner with a 40× objective.

### Thioflavin S immunofluorescence

Sections from the hippocampal/parahippocampal region were cut from all cases and were incubated with 0.1% Thioflavin-S (Sigma) solution for 7 min. The sections were differentiated in 70% ethanol and washed in Tris-buffered-saline. The standard immunohistochemistry protocol was applied to the sections to stain for Aβ, ABri and ADan, replacing di-aminobenzidine with TSA plus TMR system (Perkin Elmer) to provide the fluorescent signal. Sections were viewed with a Leica TCS4D confocal microscope using a 3-channel scan head and argon/krypton laser.

### Laser capture microscopy

To investigate differences in the Aβ species profile between the presubiculum and entorhinal cortex in the AD cases, ten-micron-thick frozen tissue sections were sampled from the hippocampal/parahippocampal region at the level of the lateral geniculate body from SAD cases (cases 3 and 19) and FAD cases (case 20 (*PS1* mutation) and case 23 (*APP* mutation)) (highlighted in Table 1). Sections were mounted onto PEN-membrane slides (Leica) coated with polyethylene naphthalate, fixed with 4% paraformaldehyde (PFA), and treated in formic acid for 5 min before the immunohistochemistry protocol for Aβ was performed. The Leica DM6000B laser capture microdissection (LCM) microscope was used to firstly dissect the Aβ-positive areas of the presubiculum from three sections per each case (average area per case 1.6 μm^2^) and 300 amyloid plaques (average area per case 2.0 μm^2^) from the neighbouring entorhinal cortex. Samples were collected from both regions for matrix-assisted laser desorption/ionization-time-of-flight (MALDI-TOF) mass spectrometric analysis to identify the Aβ peptides present in both brain regions. Samples were also collected from both brain regions via LCM to investigate the whole proteome.

### Mass spectrometry

To identify the Aβ peptides in the laser-captured presubiculum lesions and amyloid plaques from entorhinal cortex samples were aspirated with 70% formic acid, centrifuged and aspirated again. Samples were vortexed and dried before being resuspended in 5 μl 0.1% FA/20% acetonitrile (ACN). To prepare the matrix, 0.5 μl of the seedlayer (20 g/L α-cyano-4-hydroxycinnamic acid (CHCA) in 90% acetone/10% methanol with 0.005% trifluoroacetic acid [TFA]) was added to the probe. An aliquot sample (2 μl) was mixed with 1 μl sample matrix [15 g/L CHCA] in ACN and 0.1% TFA [1:1]) before being placed on the probe. The different Aβ peptides in each sample were determined using MALDI-TOF-MS [56].

To investigate the proteome in the laser-captured presubiculum and entorhinal cortex, samples were homogenised in 50 mM Ambic buffer with 2% ASB-14 using the Precellys 24 homogenizer (Bertin Instruments). The total protein concentration was determined by BCA protein assay (Thermofisher). Due to the low amount of protein present in the laser captured material samples had to be pooled. A sample pool of each disease group was created with equal protein concentration from each case. Samples were fractionated to look at soluble and insoluble proteins. Samples were spun at maximum speed for ten minutes at 4 °C and the supernatant containing the soluble proteins was removed. The remaining pellet was resuspended in ice cold acetone, vortexed and left at − 20 °C for at least an hour. Samples were vortexed and spun at 14000 g for ten minutes at 4 °C, the supernatant was removed. The pellets containing the insoluble proteins were air-dried and re-suspended in 70% formic acid and vortexed and dried in a speed-vac. Ice-cold acetone was added to the original soluble protein supernatants and left at − 20 °C overnight. The supernatants were vortexed and spun at 14000 g for ten minutes at 4 °C. The supernatant was removed and the remaining pellet was dried. Dried supernatants and pellets were reconstituted in 100 mM Tris/8 M Urea/2% ASB-14, pH 7.79, vortexed and left shaking for one hour to resolubilise the proteins. Dithioerythritol (30 mg/ml, Sigma) was added, vortexed and left shaking for a further hour as a reducing agent. To prevent the formation of disulphide bonds iodoacetamide (36 mg/ml, Sigma) was added to alkylate the proteins, before the addition of H_2_O. 1 μg Trypsin-LysC enzyme (Promega Trypsin/Lys-C Mix, Mass Spec Grade cat no. V5073) was added to digest the proteins and was left for three-four hours at 37 °C. The digested samples were diluted 1:1 in 0.2% TFA and 5 pmol of Waters MassPREP enolase digestion standard (part no. 186002325) was added to the digest. Peptides were cleaned in Agilent C18 Bond Elut 96 well plates (Part no. A496011C). The wells were primed with 50% ACN/0.1% TFA and then washed twice with 0.1% TFA. The diluted digest samples were added to the wells and allowed to drip through. The residual salts were washed away using 3% ACN/0.1% TFA and peptides eluted using 2 × 50% ACN/0.1% TFA.

The digested cleaned peptides were reconstituted in 3% ACN/0.1% TFA. MS^e^ Label-free mass spectrometry was performed as previously described [6] with a SYNAPT G2-Si High Definition machine with 2D fractionation. Four fractions were run for each sample and 0.5 μg of protein was injected per fraction per run. The order of samples were randomised to avoid technical bias. The raw data was processed using Progenesis QI for proteomics (Nonlinear Dynamics, UK). Peptides were identified with an MS^e^ search against the UniProt reviewed human proteome with the following parameters: missed cleavages, max one; max protein mass, 800,000 kDa; modifications of carbamidomethyl C, oxidation M, demidation N and Q. The ion matching requirements were set as follows: three fragments/peptide, five fragments/protein, one peptide/protein. Proteins that had been identified with only one unique peptide were excluded from the analysis. Protein identification and normalised abundance was exported into excel for further bioinformatics.

### Bioinformatics

Proteins that had > 1.5-fold change in expression between regions were put into publicly available databases to assess the biological processes, molecular functions and cell components that were enriched in each region. Webgestalt was used to assess the enriched gene ontology terms [87].

### Morphological analysis

Sequential sections were immunohistochemically stained for Aβ, AT8, Iba1, CD68 and CR3–43. Sections stained with Aβ were used to identify the ‘lake-like’ deposit in the presubiculum and entorhinal cortex in each case. Bland-Altman plots were used to determine the intra- and inter-rater reliability for the number of randomized snapshot areas required for the quantitation of the different immunohistochemical preparations [5]. Using Image J software (https://imagej.net/) and setting the threshold to include only di-aminobenzidine staining, twenty randomised snapshots from an identified region of interest, each measuring 1500 μm × 1000 μm, were generated, using a python script, from the pre-marked presubiculum and the entorhinal cortex. Images were captured using Leica Image scope. The twenty snapshots were then used to determine the mean ‘areal fraction’ for each immunohistochemical preparation, defined by the ratio of the area occupied by positive immunohistochemical staining and the field of interest [25]. Iba1, CD68 and CR3–43 immunohistochemical preparations were used for the assessment of the microglial response while AT8 immunohistochemistry was employed to determine the phosphorylated tau load, comprising NFTs, neuropil threads (NTs) and plaque-associated abnormal neurites. In addition, the number of NFTs, identified as large filamentous tau-positive structures in the neuronal cytoplasm [57], were also determined in both the presubiculum and entorhinal cortex. NFTs were systematically counted with any partial NFTs being excluded.

### Statistical analysis

Statistical analysis was performed using GraphPad Prism 5 software (GraphPad Software Inc. USA). Continuous variables were analysed using either a two-tailed *t*-test or a Mann-Whitney U test, as appropriate while continuous variables between several groups were compared using the Kruskal-Wallis test. The statistical significance level was established at *p* < 0.05.

## Results

### Comparison of the pathological characteristics in the presubiculum and entorhinal cortex in AD, FDB and FDD

**Fig. 1:**
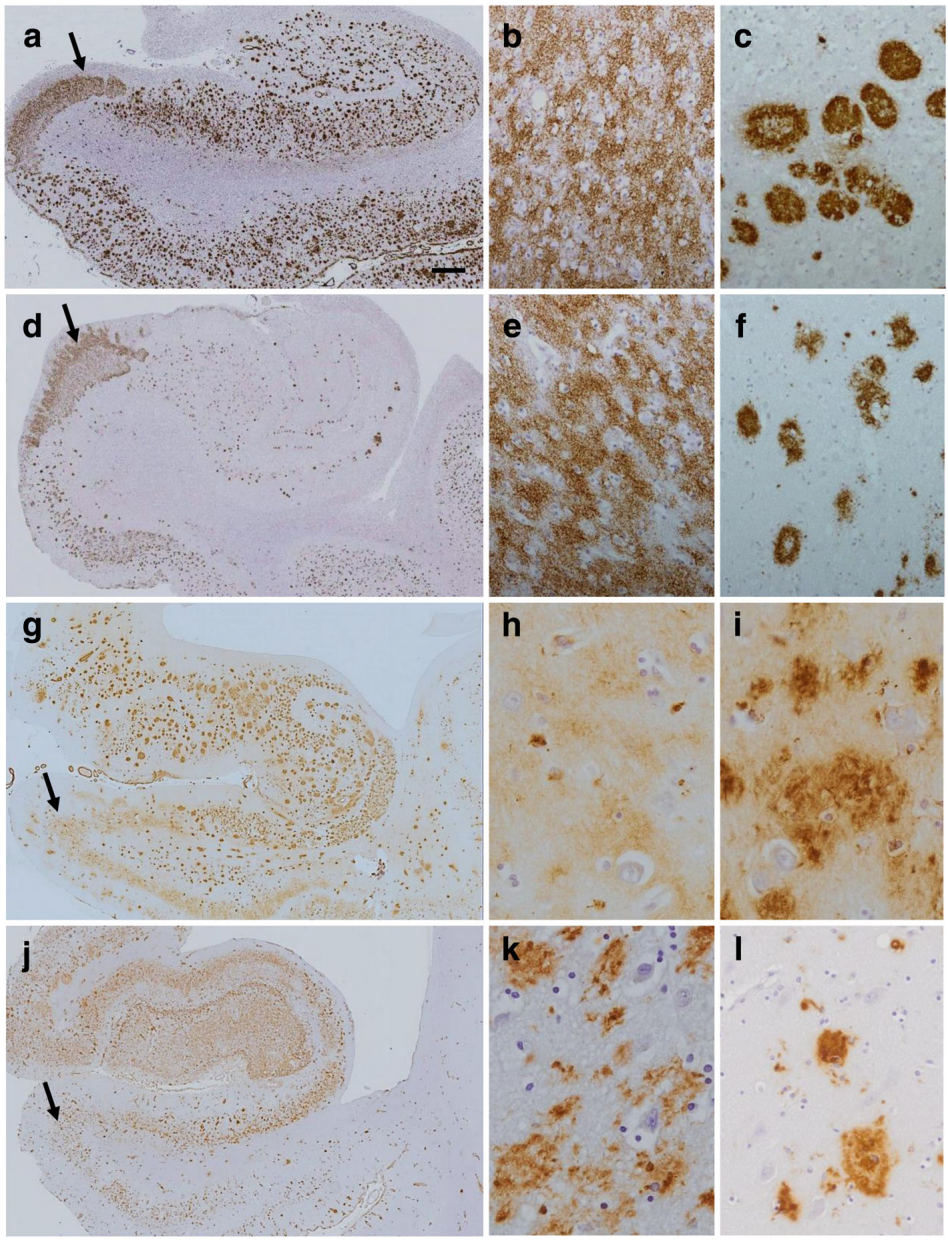
Pathological analysis of the presubiculum in familial and sporadic Alzheimer’s disease (AD; **a**-**f**), familial British dementia (FBD; **g**-**i**) and familial Danish dementia (FDD; **j**-**l**). Aβ immunohistochemistry demonstrates large diffuse, ‘lake-like’ deposits in the presubiculum in both familial AD (case 26; **a**, arrow; **b**, presubiculum at higher magnification) and sporadic AD (case 2; **d**, arrow; **e** presubiculum at higher magnification). In both disease types well-defined Aβ plaques were present in the entorhinal cortex as shown in sporadic AD (**c** and **f**). The ABri-positive (case 31; **g**-**i**) and ADan-positive (case 36; **j**-**l**) parenchymal deposits show similar morphological patterns in FBD and FDD, respetively. Bar in a represents 1000 μm in **a**,**d**,**g**, and **j**; 50 μm in all remaining images

The pathology observed in the presubiculum was morphologically different from that in the entorhinal cortex in all cases of SAD and FAD investigated (Fig. 1). In the presubiculum Aβ deposited as hazy, diffuse ‘lake-like’ lesions in the parenchyma (Fig. 1a and d arrows and 1b and 1e, higher magnification) whereas large numbers of ‘mature’ Aβ amyloid cored plaques with well-defined edges were present in the entorhinal cortex (Fig. 1c and f). A similar difference in the morphology of the parenchymal deposits between these two anatomical areas was observed in two other cerebral amyloid diseases, FBD and FDD. Accordingly diffuse, ‘lake-like’ ABri and ADan deposits were seen in the presubiculum (Fig. 1g and j, arrows, shown at higher magnification Fig. 1h and k) and well-defined amyloid plaques in the entorhinal cortex (Fig. 1i and l) of FBD and FDD, respectively.

**Fig. 2:**
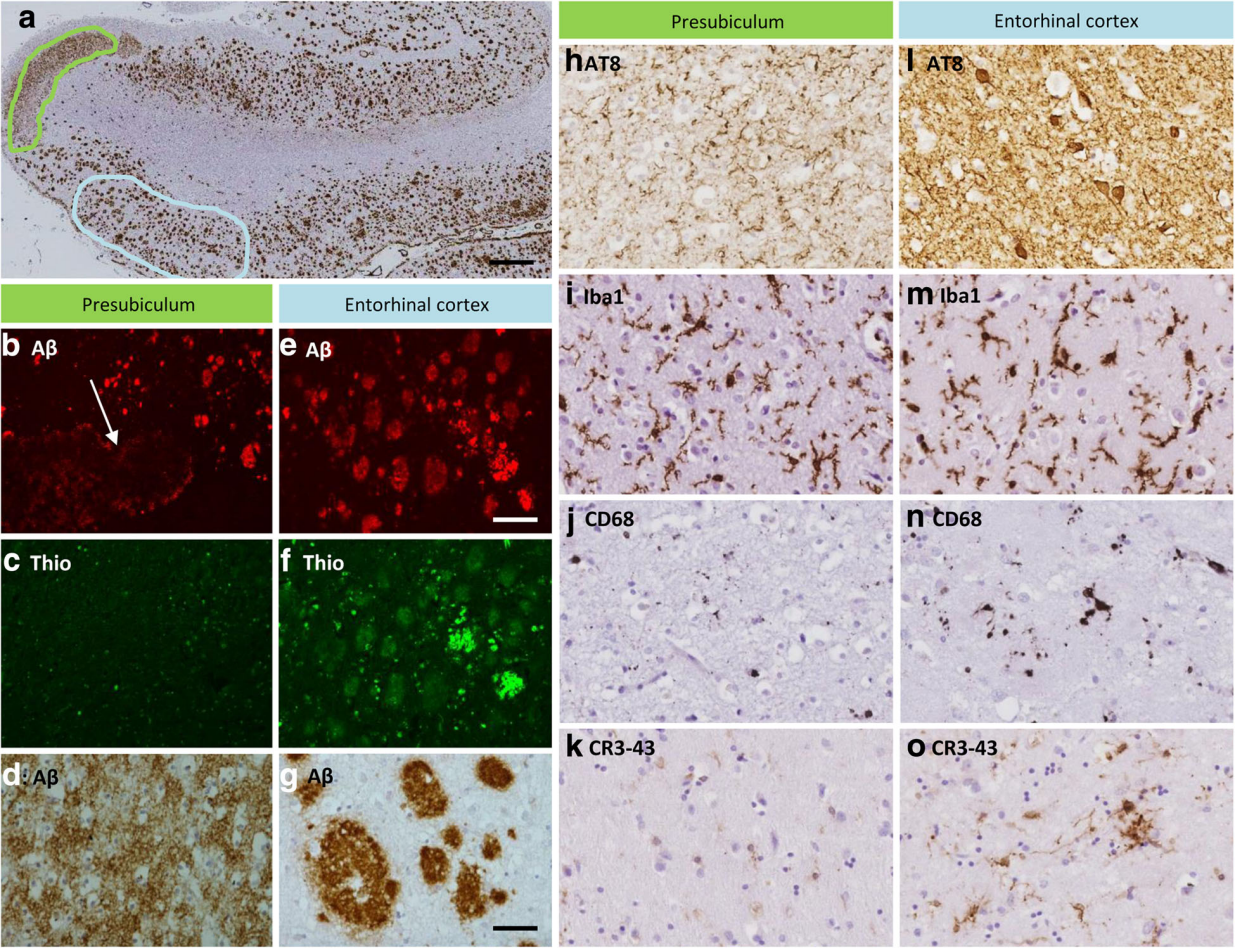
Pathological comparisons of the presubiculum and entorhinal cortex in Alzheimer’s disease. The image demonstrates the anatomy of the hippocampus and illustrates the difference in Aβ deposition between the presubiculum (green outline) and entorhinal cortex (blue outline). Fluorescent Aβ immunohistochemistry shows that the Aβ peptide is deposited in a diffuse manner in the presubiculum (**b**, white arrow) whereas defined Aβ plaques are shown in the entorhinal cortex (**e**). Thioflavin S staining highlights the Aβ plaques in the entorhinal cortex (**f**), whereas the presubiculum is negative for the Thioflavin S stain demonstrating the Aβ in the presubiculum contains pre-amyloid deposits (**c**). Tau immunohistochemistry shows a difference between the presubiculum (**h**) and entorhinal cortex (**l**) in the density of neuropil threads and neurofibrillary tangles. The microglial marker, Iba1, shows the total number of microglia being equal between the two regions (**I** and **m**), whereas CD68 and CR3–43 highlight the increase in the number of activated microglia in the entorhinal cortex (**n** and **o**) compared to the presubiculum (**j** and **k**). Bar in ‘a’ represents 1000 μm in **a**; 100 μm in **b**, **c**, **e**, and **f**; 50 μm in **d**, **g** and **h**-**o**

Aβ, ABri or ADan immunohistochemistry combined with Thioflavin-S staining revealed that the extracellular proteins found in the lake-like peptide deposits of the presubiculum (Fig. 2b, arrow) were Thioflavin-S negative in all cases (Fig. 2c) indicating that such deposits were of pre-amyloid nature in both SAD and FAD. In contrast, the plaque-like lesions of the entorhinal cortex were positive for both Aβ (Fig. 2e) and Thioflavin S (Fig. 2f) indicative of amyloid conformation of the Aβ species.

### Tau deposition and NFT frequency in FAD and SAD

**Fig. 3:**
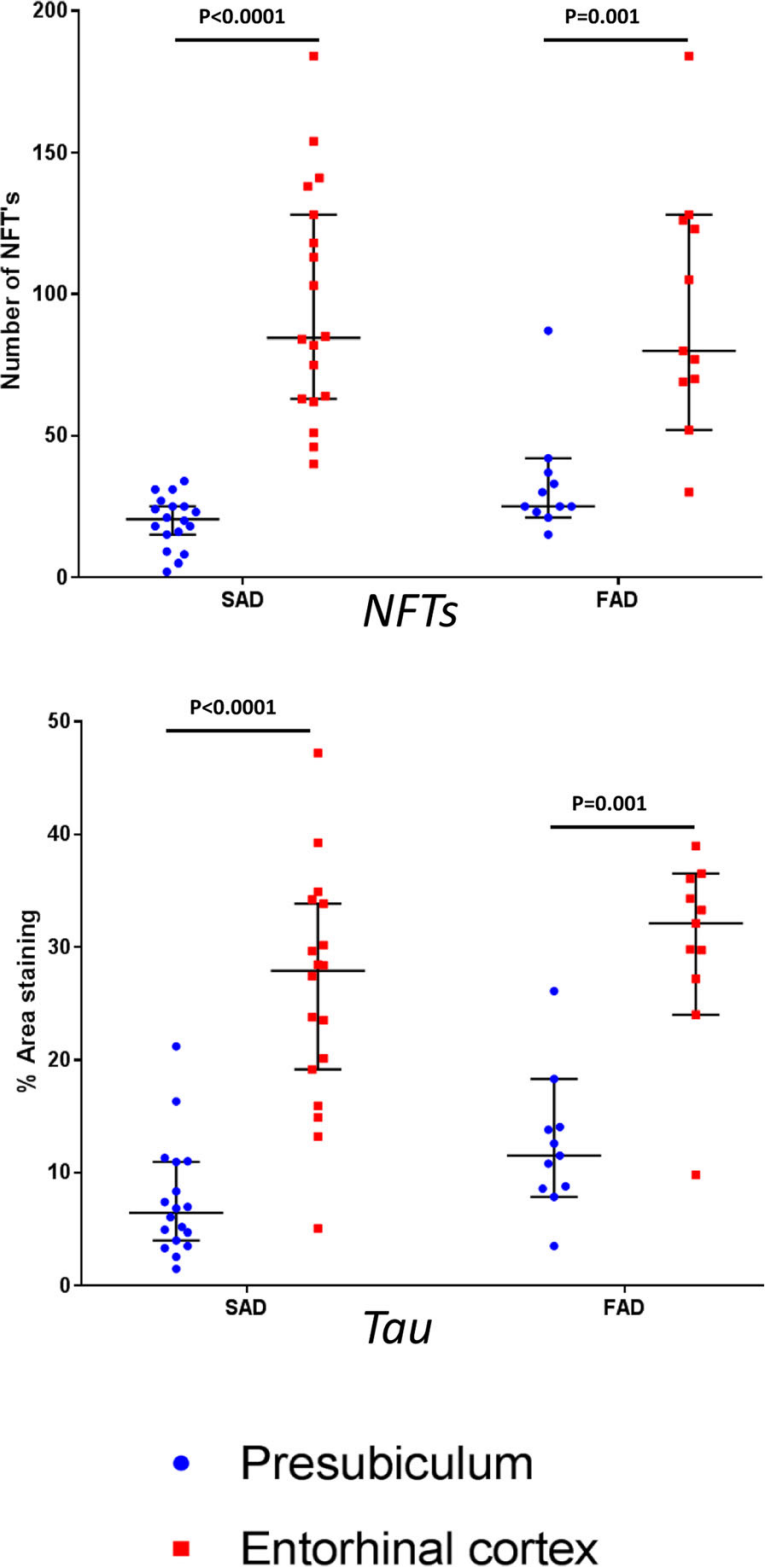
Quantification of microglial immunohistochemistry in the presubiculum and entorhinal cortex**.** The overall numbers of microglia were similar in the two anatomical areas as shown by the Iba1. However the presubiculum had significantly less activated microglia, positive for CD68 and CR3–43, than the entorhinal cortex in both sporadic and familial AD cases. FAD; Familial Alzheimer’s disease: SAD; Sporadic Alzheimer’s disease. Bars represent medians with 95% confidence intervals. Wilcoxon paired rank test was performed in each case and *p* values are shown

The phosphorylated tau load comprising all tau-positive lesions was significantly greater in the entorhinal cortex compared to the presubiculum in both SAD (*p < 0.0001*) and FAD (*p = 0.001*) (Table 2; Figs. 2h, l and 3). Furthermore, there were significantly more NFTs in the entorhinal cortex compared to the presubiculum in both AD groups **(**SAD: *p < 0.*0001; FAD: *p = 0.001*) (Table 2; Figs. 2h, l and 3).

**Table 2:** Statistical analysis of the immunohistochemical quantification

		AT8		NFT’s		Iba1		CD68		CR3–43	
		Presubiculum	Entorhinal cortex	Presubiculum	Entorhinal cortex	Presubiculum	Entorhinal cortex	Presubiculum	Entorhinal cortex	Presubiculum	Entorhinal cortex
SAD	Mean	11.30%	41.20%	15	41	4.10%	3.40%	1%	1.80%	2.50%	5.90%
	Range	0.9–35.6%	1.9–81%	8–27	12–73	0.3–8.9%	0.5–8.9%	0.1–2.6%	0.4–6.1%	0.2–18.6%	1–44.9%
	SD	9.50%	23.80%	6	16	2.70%	2.60%	0.90%	1.50%	4.10%	9.80%
FAD	Mean	13.30%	42.20%	16	34	5.60%	5.30%	1%	1.60%	2.20%	4.10%
	Range	1.3–26.3%	3.1–68.9%	3–32	2–68	0.9–17.9%	2–9.6%	0.6–2.2%	0.7–5%	0.1–8.6%	0.2–18.6%
	SD	9.10%	24.30%	10	23	5.10%	2.30%	0.50%	1.30%	2.60%	5.40%

Table shows the mean percentages, range and standard deviation of the immunhistochemical analysis using AT8, to quantitate the numbers of NFTs and total phosphorylated tau load; Iba1, CD68 and CR3-43 to quantitate the numbers of microglia in both the presubiculum and entorhinal cortex

### Microglial activation in FAD and SAD

**Fig. 4:**
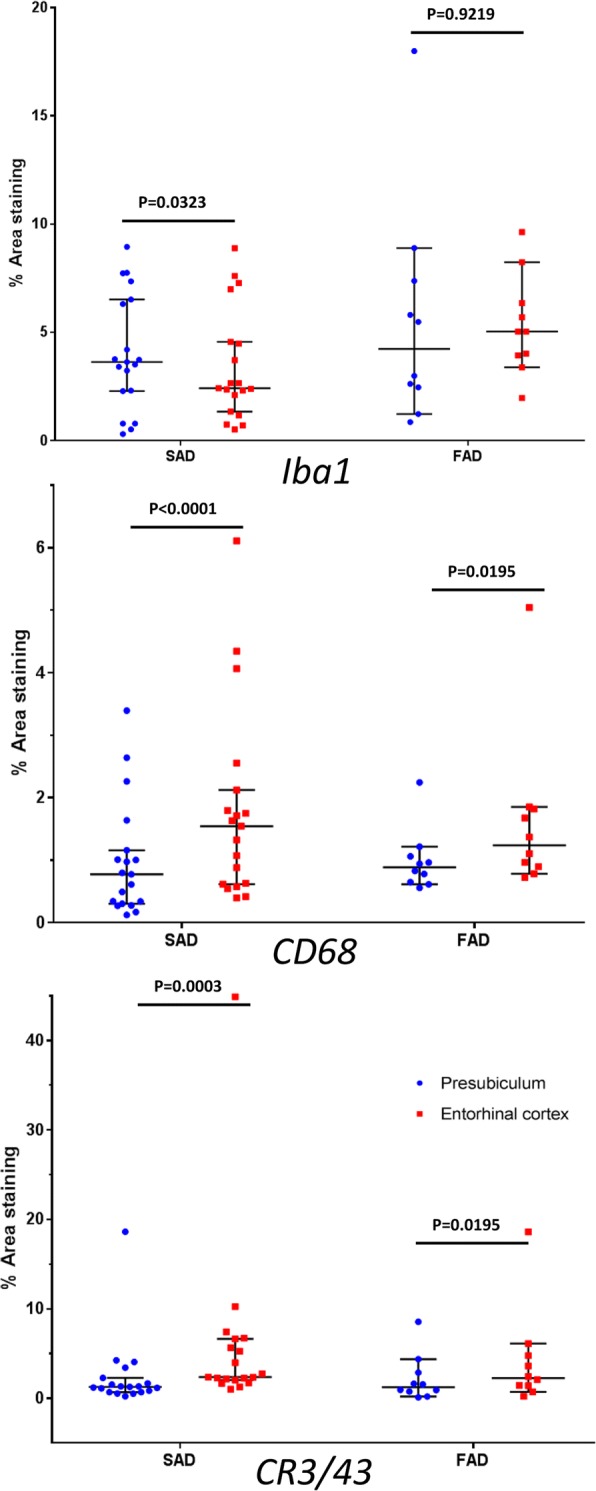
Quantification of tau immunohistochemistry in the presubiculum and entorhinal cortex. The density of the tau immunohistochemistry observed in the entorhinal cortex and presubiculum was quantitated. The overall density included NFTs and neuropil threads was significantly higher in the entorhinal cortex compared to the presubiculum. The number of NFTs was also significantly higher in the entorhinal cortex compared to the presubiculum. FAD; Familial Alzheimer’s disease: SAD; Sporadic Alzheimer’s disease. Bars represent medians with 95% confidence intervals. Wilcoxon paired rank test was performed in each case and *p* values are shown.

Neuroinflammation is a prominent feature of neurodegenerative diseases. In this study we assessed the extent of neuroinflammatory response by determining the density of three microglia markers in the presubiculum and entorhinal cortex. The Iba1 marker, a calcium binding protein that detects total numbers of microglia, was used to assess any homeostatic microglia present [8, 69]. The CD68 and CR3–43 antibodies both detect activated microglia, phagocytic microglia and antigen presenting microglia, respectively [8, 39]. The density of the resident microglia, as detected by Iba1 immunohistochemistry, was similar in the presubiculum and the entorhinal cortex in FAD (*p = 0.92)* (Table 2). Whereas a similar analysis in SAD showed that more microglia were present in the presubiculum than in the entorhinal cortex (*p = 0.03*) (Table 2; Figs. 2i, m and 4). However, CD68 (*p < 0.0001* and *p = 0.02* in SAD and FAD respectively) and CR3–43 (*p = 0.0003* and *p = 0.02* in SAD and FAD respectively) preparations showed that the area density of the microglia was sig nificantly reduced in the presubiculum compared with the entorhinal cortex in both the SAD and FAD groups (Table 2; Figs. 2j-o and 4).

### Identification of Aβ species in FAD and SAD

**Fig. 5:**
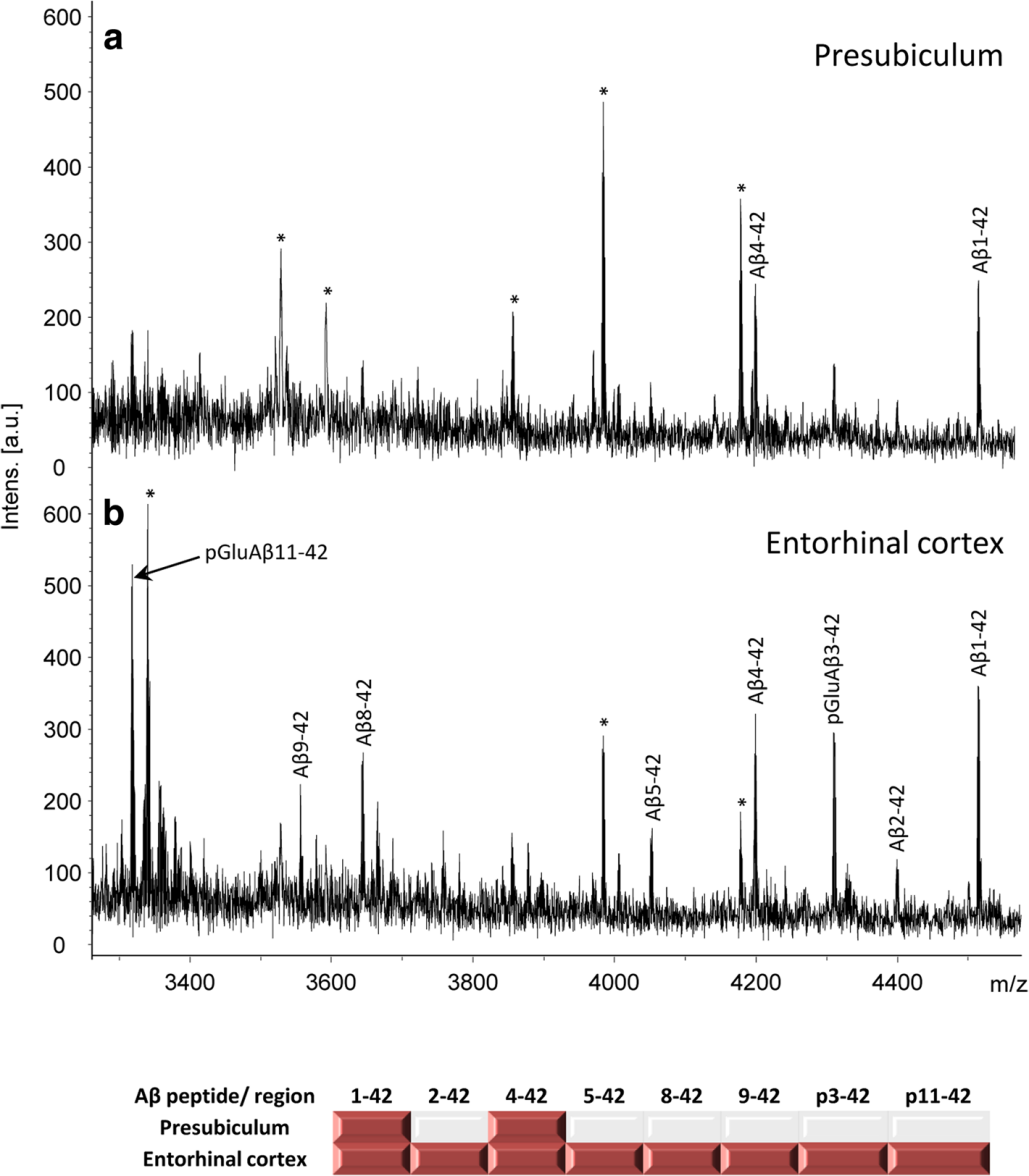
Mass spectra of the Aβ peptide pattern from the presubiculum and entorhinal cortex. Representative mass spectra from case 3 highlights the differences between the full length Aβ species identified in the presubiculum (**a**) compared to the truncated and post-translationally modified Aβ species identified in the entorhinal cortex (**b**).The table shows the Aβ isoforms present in the presubiculum and entorhinal cortex from six Alzheimer’s disease cases. * represent unidentified non-Aβ related peaks. A peak was considered as identified if the signal-to-noise was > 2

**Fig. 6:**
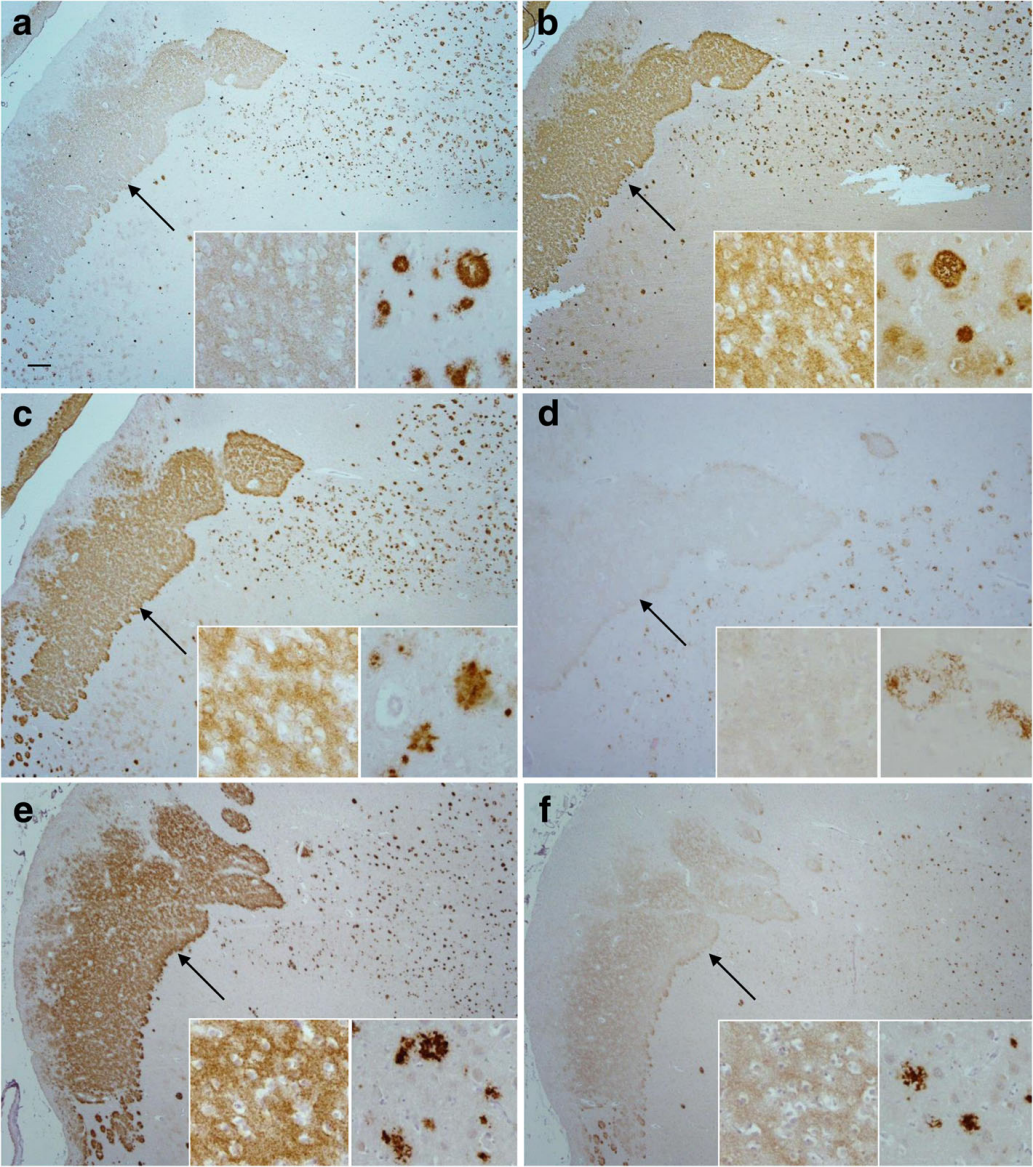
pGlu-Aβ immunohistochemistry in the presubiculum and entorhinal cortex Immunohistochemical analysis of pGlu truncated Aβ species was carried out with four antibodies Aβ-pE3 (**a**; Synaptic systems 218,311; clone 1–57); Aβ-pE3 (**b**; Synaptic systems 218,011; clone 2–48); Aβ-pE3 (**c**; Synaptic systems 218,003); Aβ-pE11 (**d**; Synaptic systems 218,811; clone 173D8); Aβ4-x (**e**; [83]; Aβ1–5 (**f**; Synaptic systems 218,231). All antibodies showed a degree of positive staining in both the presubiculum and the entorhinal cortex. Bar in A represents 250 μm in a, b, c and d and 20 μm in the inserts

LCM and MALDI-TOF-MS were utilised to examine whether the biochemical profile of the Aβ species found in the presubiculum were different from species present in amyloid plaques isolated from the entorhinal cortex. These studies showed no difference in the profile of the Aβ peptide species between the SAD and FAD cases (Fig. 5). Full length Aβ_1–42,_ numerous N-terminally truncated peptides and post-translationally modified peptide Aβ species with pyroglutamate at positions 3 or 11 were identified in the entorhinal cortex. This was in contrast to the Aβ peptides identified in the presubiculum where full length Aβ_1–42_ and the N-terminally truncated Aβ_4–42_ were identifed. No pyroglutamate post-translationally modified Aβ species were identified in the presubiculum in either SAD or FAD (Fig. 5). To validate the differences in Aβ species present in the entorhinal cortex compared to the presubiculum immunohistochemical methods utilising antibodies raised against N-terminally truncated and pyroglutamate modified Aβ species were employed. All antibodies (1–57 N-terminal Aβ_pE3_, 2–48 N-terminal Aβ_pE3_, pE-Aβ, Aβ-pE11, Aβ4-x, Aβ1-x) labelled the Aβ plaques in the entorhinal cortex and some degree of positve staining in the presubiculum was observed (Fig. 6a-f). Positive immunohistochemical staining for these Aβ species in the presubiculum indicates that the Aβ peptides are present in the presubiculum but the amount may differ between the two regions, making them undetectable by MALDI-TOF MS or that the species specific antibodies might show some minor cross-reactivity with other Aβ species (e.g. unmodified Aβ3–42) [85].

### Proteomic expression in the presubiculum in FAD and SAD

We investigated alterations in protein expression in the presubiculum compared to the entorhinal cortex using MS^e^ label free proteomics. Samples were analysed as soluble protein supernatant and insoluble protein pellet fractions with a total of 561 proteins detected in the supernatant and 1824 proteins in the pellet. Some proteins were detected in both fractions (303 proteins) or only in one (supernatant 254 proteins, pellet 1488 proteins). SAD cases were pooled into one sample and FAD cases pooled into another. Due to pooling the samples, statistics could not be undertaken, instead fold change between regions was determined to highlight the greatest changes occurring. The fold change expression was calculated for each protein for the presubiculum compared to the entorhinal cortex. SAD and FAD cases were analysed separately and additionally, an average fold change between both was calculated. When analysing the average fold change between SAD and FAD, a total of 112 proteins had an increased fold change of 1.5 or greater in the presubiculum compared to the entorhinal cortex in the supernatant and 313 in the pellet (Additional file 1: Table S1 and Additional file 2: Table S2). Proteins were also found to be decreased in the presubiculum compared to the entorhinal cortex in the supernatant (130 proteins) and the pellet (703 proteins) (Additional file 3: Table S3 and Additional file 4: Table S4). Several proteins were markedly increased in the presubiculum or entorhinal cortex at a level above 1.5-fold, highlighting proteomic differences between the two regions. Webgestalt gene ontology analysis of the proteins with > 1.5-fold change in expression highlighted the top biological processes enriched at a level of *p* < 0.05 (Additional file 5: Table S5 and Additional file 6: Table S6). The proteins that were shown to increase in the presubiculum compared to the entorhinal cortex were enriched for biosynthetic and biogenetic processes as well as metabolic changes. However, the proteins that were shown to decrease in the presubiculum compared to the entorhinal cortex were enriched for cell organisation and cell signalling.

Among the proteins identified as altered between the two brain regions, we found a proportion previously reported as AD-associated proteins. These included proteins associated with amyloid processing and fibrillisation, tau accumulation, and inflammation such as SNX6 and RAB21 which were only detected in the entorhinal cortex and S100A8 and OTUB1 were decreased in the presubiculum (14.8-fold and 2-fold respectively). SNX6 negatively regulates BACE1 cleavage of APP [54]. RAB21 overexpression results in greater Aβ production, whereas silencing of this gene reduces the Aβ levels [70]. S100A8 is thought to aggregate itself prior to Aβ plaque formation and treatment of a neuronal cell line with S100A8 leads to increased Aβ1–42 but decreased levels of Aβ1–40 [45]. Furthermore, S100A8 forms a heterodimeric complex with S100A9 molecules which then elicits an inflammatory response. S100A9 is increased in the presubiculum in this data set, whereas S100A8 was decreased [19, 29]. Therefore we can hypothesis that this complex is not active in the presubiculum [36, 37]. OTUB1 (OTU Deubiquitinase, Ubiquitin Aldehyde Binding 1), a tau deubiquitinating enzyme, has been shown to increase AT8 positive tau accumulation and to increase tau-seeded tau aggregation [82].

Inflammatory proteins detected included DOCK2, decreased 9.4 fold in the presubiculum and INPP5D, increased 2.2-fold in the presubiculum. DOCK2 is part of the prostaglandin pathway and has been shown to modulate microglial cytokine secretion and phagocytosis [12]. INPP5D is a negative regulator of the innate immune system. It has been linked to AD in GWAS studies [18, 24, 49]. Additionally, multiple annexins showed protein expression changes between regions. ANXA4 (329.9 fold, pellet), ANXA7 (4.3 fold, pellet), ANXA1 (2 fold, pellet), ANXA6 (1.7 fold, pellet), ANXA5 (1.4 fold, pellet, 1.2 supernatent), ANXA11 (1.4 fold, pellet) and ANXA2 (1.03 fold, pellet) were found to be decreased in the presubiculum. The annexins are a multigene family that are Ca^2+^ and phospholipid binding proteins. They have a range of roles including involvement in inflammation. Annexin A1 promotes resolution of inflammation by supressing microglial activation and inhibiting secretion of their pro-inflammatory cytokines [59]. Annexin A2 has been shown to modulate pro-inflammtory cytokines and reactive oxygen species [26]. Annexin A5 inhibits phagocytosis and also regulates cytokine secretion and Annexin A6 has a role in T cell proliferation [14, 62].

### Immunohistochemical analysis of candidate proteins in AD

**Fig. 7:**
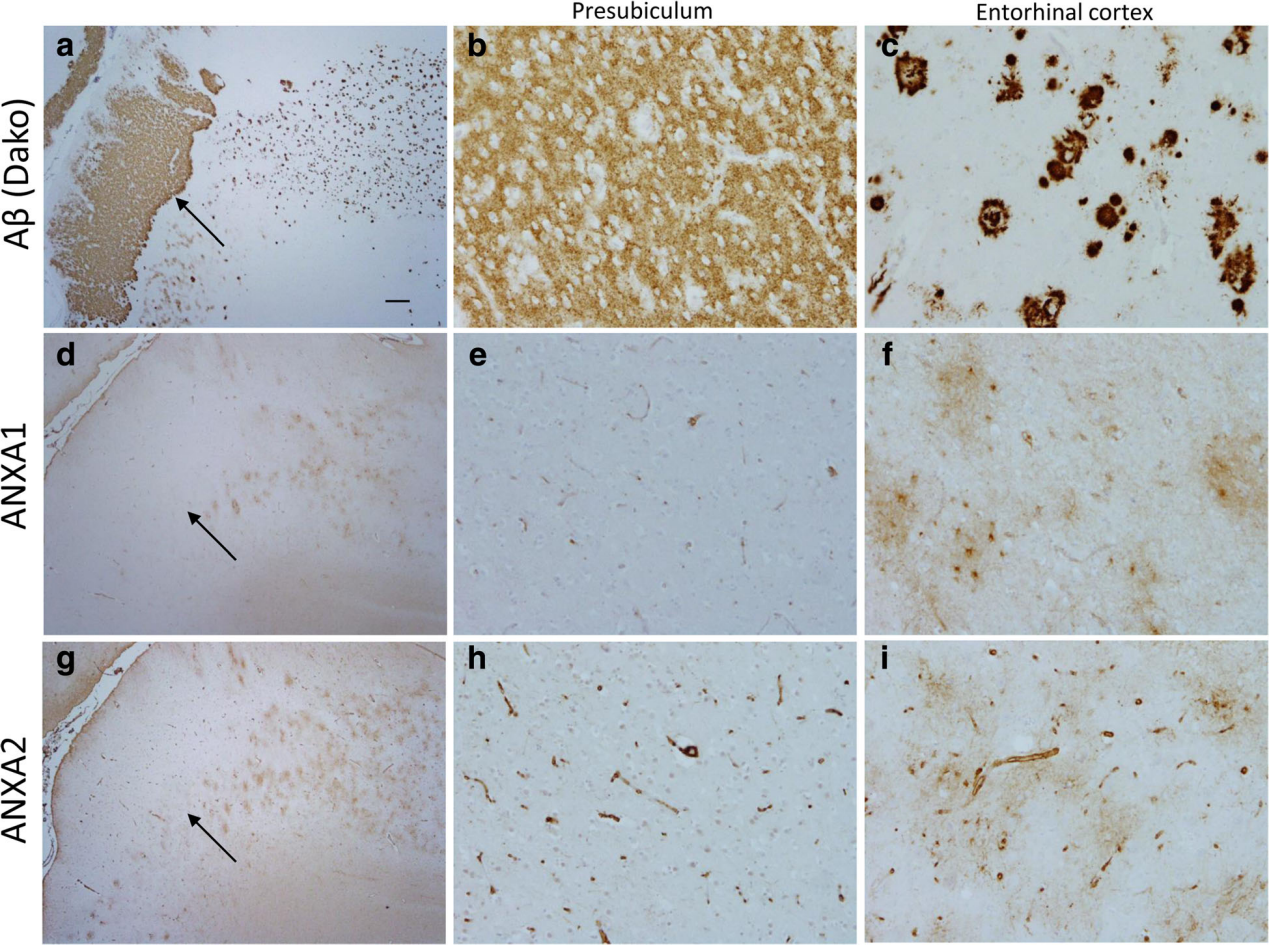
Immunohistochemical analysis of annexins in the presubiculum and entorhinal cortex. Immunohistochemical analysis of Annexin A1 (**d**-**f**) and Annnexin A2 (**g**-**i**) compared with Aβ deposition (**a**-**c**). Serial sections were immunostained for Aβ to identify the ‘lake-like’ Aβ deposit found in the presubiculum (arrows), with image of higher magnification showing the different Aβ morphologies in the presubiculum (**b**) and entorhinal cortex (**c**). Positive Annexin A1 and Annexin A2 staining was observed in the entorhinal cortex (**f** and **i**) compared to a lack of positive staining in the presubiculum (**e** and **h**). Bar in A represents 250 μm in **a**, **d** and **g** and 50 μm in rest of panels

To validate the proteins of interest detected by MS^e^ label free proteomics, immunohistochemical analysis was performed on the hippocampal sections containing the presubiculum and entorhinal cortex. Proteins to validate were chosen based on the proteomic data, published literature and commercially available antibodies previously used with paraffin embedded tissue. To assess the effect on inflammation in the presubiculum one protein that was increased in the presubiculum (DOCK2), one protein that was decreased in the presubiculum (INPP5D). The annexin protein family were also significantly changes between the two brain regions and two annexins (ANXA1 and ANXA2) were investigated using commercially available antibodies. DOCK2, and INPP5D did not show any positive staining in the presubiculum or the entorhinal cortex. However ANXA1 and ANXA2 antibodies stained astrocytes and astrocytic processes in the entorhinal cortex. However there was no staining in the presubiculum (Fig. 7), validating the proteomic results obtained in the pellet by mass spectrometry.

## Discussion

This study aimed to investigate the pathological features in the morphologically diverse presubiculum and entorhinal cortex in three cerebral amyloidosis. We have shown that, regardless of the amino acid sequence of the three amyloid peptides Aβ, ABri and ADan they form similar diffuse, ‘lake-like’ structures in the presubiculum in AD, FBD and FDD respectively. This observation underpins the notion that this morphological phenomenon of the presubiculum is not a unique feature of the Aβ peptide of AD, but is also seen in FBD and FDD. The lack of binding to β-pleated sheet ligands, such as Thioflavin-S, implies that the large diffuse ‘lake-like’ deposits seen in the presubiculum of the three cerebral amyloid diseases are primarily composed of non-fibrillar peptide aggregates [2, 30, 31, 84]. A recent study on binding of amyloid tracers to Aβ aggregates showed no binding of the PiB PET ligand in the presubiculum of AD patients, also indicating that the Aβ deposits found in the presubiculum are not in amyloid conformation [36].

It has been suggested that diffuse plaques with non-fibrillar Aβ undergo fibrillization and become amyloid/neuritic plaques [13, 33, 46, 55, 57]. However, the large diffuse, ‘lake-like’ Aβ deposits of the presubiculum do not progress into mature amyloid plaques. Considering the phases of Aβ deposition in AD, diffuse plaques appear in the presubiculum in Thal phase 2 in around one third of the cases to be present in all cases by Thal phase 3. The morphology of such ‘lake-like’ deposits remains unaltered during progression of the amyloid pathology from Thal phase 3 to Thal phase 5 indicating that these large Aβ deposits do not fibrillise into amyloid [74].

In this study further biochemical and proteomic analysis was carried out in the AD cases to understand the morphological differences between the presubiculum and the entorhinal cortex. Biochemical analysis was carried out in a limited number of cases, however the results were conclusive for each case and the proteomic investigations were carried out as preliminary investigations to understand difference in molecular pathways between the two brain regions. In this study we identified Aβ_1–42_ and Aβ_4–42_ in the presubiculum and no Aβ peptides terminating at residue 40 with mass spectrometry, whereas plaques isolated from the entorhinal cortex contained a mixture of Aβ peptides of various lengths with pyroglutamate modifications at positions 3 and 11. The Aβ peptides are generated by proteolytic cleavage of the larger amyloid precursor protein (APP) by β- and γ-secretase [22, 27, 67]. While a single protein BACE1 is responsible for the β-secretase activity, γ-secretase is composed of four essential subunits: presenilin 1 (PS1) or presenilin 2 (PS2), together with nicastrin, APH-1 and PEN-2 [53, 76]. The γ-secretase complex cleaves at multiple sites within the transmembrane domain of APP, generating Aβ peptides ranging in length [66]. It is known that the distribution of these species varies among different pathological lesions. Parenchymal deposits consist of Aβ_42_ as the major component, whereas Aβ deposits within vessels are mainly Aβ_40_ [61]. The reasons and relevance of this selectivity remains unclear. The biophysical and biochemical properties of Aβ vary immensely with its length, the longer Aβ_42_ has a much greater tendency to aggregate than the shorter Aβ_40_ [34, 35]. Furthermore the relative ratio of Aβ_40_ to Aβ_42_ influences the biological effects of the Aβ mixture in vivo and in vitro even when total amounts of Aβ are kept equal [40] suggesting that the ratio of Aβ_40_/Aβ_42_ is more important than the absolute amounts of Aβ_42_ [72]. The ratio also influences the morphological phenotype of the underlying pathology; by increasing the amount of Aβ_42_ the pathology shifts from predominantly CAA to parenchymal plaques [28]. Post-translational modifications of Aβ result in N-terminally truncated Aβ with pyroglutamate modifications at position 3 (Aβ_N3pE_) or 11 (Aβ_N11pE_). Pyroglutamate modification is associated with enhanced aggregation into oligomers and fibrils. Biochemically, Aβ_42_ is the first Aβ species to accumulate in the human brain [33, 44]. Aβ_40_ is detected subsequently, followed by N-terminal truncated and pyroglutamate-modified Aβ_N3pE_ and/or Aβ_N11pE_. These modified forms of Aβ are frequently detected in plaques of all AD cases [33, 44, 60]. The Aβ species detected by mass spectrometry in the presubiculum showed no pyroglutamate modifications, suggesting that the Aβ peptides found in the presubiculum are not modified overtime. However, we could detect pyroglutamate modified peptides when using pGlu specific antibodies. This may indicate that relatively small concentrations of these peptides are found in the presubiculum. It is also of note that the FAD cases investigated in this study carried mutations in the *APP* and *PSEN1* genes, both mutations resulting in the production of different amounts of the Aβ variants. Yet all cases regardless of mutation status have the same characteristic morphology, and mass spectrometry profile of Aβ in the presubiculum. N-terminally truncated and pyroglutamate-modified Aβ peptides have previously been shown in dense amyloid plaques, so lower quantities in the presubiculum may suggest that these modifications of Aβ are required for amyloid fibrils to form.

Although the contribution of the canonical α-, β- and γ-secretases to APP processing have been studied in depth, the proteolytic cleavage of APP may be more complex. An increasing number of additional secretases have been identified that also proteolytically process APP [3]. However, further investigations on the underlying secretases involved in the processing and cleavage of Aβ are needed to determine whether other secretases or related proteins are found in the presubiculum compared to the entorhinal cortex [11, 71]. This though would not account for the large diffuse protein deposit composed of ABri or ADan in FBD and FDD as these proteins are not cleaved by secretases [41]. A link between BRI2 and APP has been demonstrated; where BRI2 has been shown to specifically interact with APP. As a result of this interaction BRI2 masks the cleavage sites of β- and α-secretase on APP and the γ-secretase docking site on the APP C-terminal fragment C99. Thus, BRI2 modulates APP processing by inhibiting Aβ formation and its deposition properties [20, 47, 48]. FBD and FDD are due to mutations in the *BRI2* gene, and both mutations cause the precursor protein to be extended and through a furin-like processing two 34 amino acid peptides are generated (ABri and ADan respectively). ABri molecules have a higher tendency to form ordered oligomeric assembles than Aβ_42_ [63]. Therefore the morphological phenomenon observed in the presubiculum of three neurodegenerative diseases caused by the deposition of three different proteins would suggest that the observed phenomenon would likely be due to local tissue factors in the presubiculum rather than the processing of the different proteins.

It has previously been shown that despite the accumulation of Aβ in the presubiculum, neurofibrillary degeneration is minimal [2, 7, 21, 37], whereas the neighbouring entorhinal cortex and subiculum had considerable numbers of NFTs [7]. We have quantitated these findings in this study and found significantly more NFTs in the entorhinal cortex compared to the minimal number of NFTs found in the presubiculum. Previous studies have demonstrated that there is no neuronal loss in the presubiculum in AD compared to normal controls [21], showing that although neurons are present significantly fewer NFTs form in the presubiculum.

In the healthy brain microglia actively support neurons through the release of nerve growth factors [15, 50, 78] and have been shown to carry out many activities including synaptic pruning [1, 65]. The classification of microglia into pro-inflammatory or anti-inflammatory forms is based on the phenotypes of peripheral macrophages [38, 51, 58]. Pro-inflammatory are characterised by increased expression of pro-inflammatory mediators and cytokines [58] that retract their processes and adopt an amoeboid morphology often migrating closer to the site of injury [16]. Anti-inflammatory microglia are characterised by increased cytokine expression and associated with increased ramification of processes [58]. In the AD brain activated pro-inflammatory microglia are typically located in brain regions affected by disease and surround Aβ amyloid plaques [32]. However, the involvement of microglia in the neuroinflammatory processes is now understood to be a complex continuum rather than the originally proposed pro-inflammatory and anti-inflammatory polarised states [86]. In this study we showed a significant increase in the presence of CD68 and CR3–43 positive microglia in the entorhinal cortex compared to the presubiculum. Even though the number of microglia within the presubiculum and entorhinal cortex, as demonstrated by Iba1 staining, were not significantly different. Microglia have been proposed to assist in the clearance of Aβ from the brain [73], however this clearance by microglia is reduced in AD [43]. The lack of reactive microglia in the presubiculum would suggest that these cells are not engaged in pre-amyloid removal and are able to undertake their non-inflammatory functions specifically relating to maintenance of the central nervous system [1, 65]. Recently, Sosna et al. demonstrated that when microglia are ablated via CSF1R (Colony-stimulating factor 1 receptor) inhibition, there is a significant reduction of neuritic plaque formation and pre-fibrillar amyloid present in 5xFAD Tg mice [68], suggesting that microglia are instrumental in the formation of neuritic plaques.

We investigated alterations in protein expression in the presubiculum compared to the entorhinal cortex to assess whether a local difference in protein expression could contribute to the morphological and biochemical differences in the two brain regions. We demonstrated that 429 proteins had an increased expression in the presubiculum compared to the entorhinal cortex in both soluble and insoluble fractions whereas 850 proteins were shown to be decreased in the presubiculum. The proteins identified spanned many different biological processes from phospholipase activity and enzyme inhibitor activity being increased to neurotransmitter transport and synaptic transmission being decreased. We selected proteins for further validation based on the most prominent biological functions represented, the greatest fold change and the availability of commercially available antibodies. From the proteins selected, ANXA1 and ANXA2 both differed in staining pattern showing positive staining in the entorhinal cortex and was negative in the presubiculum. Further investigation into the annexin pathways and inflammatory mechanisms may identify why this region appears to be protected from neurodegeneration compared to its neighbouring regions.

We found that the ‘lake-like’ pre-amyloid deposits found in the presubiculum are not a unique feature in AD but is also found in other cerebral amyloidosis. Significant quantitative differences were found in the amount of tau, number of NFTs and the amount of microglial activation in the presubiculum compared to the entorhinal cortex. Proteomic differences were found in the Aβ species and the whole proteome between the two brain regions. In summary, understanding why the presubiculum has a different morphological appearance, biochemical and proteomic makeup compared to surrounding brain regions severely affected by neurodegeneration could lead us to understanding protective mechanisms in neurodegenerative diseases.

## Additional files

Additional file 1: **Table S1.** Proteins identified in supernatant with increased expression in the presubiculum compared to the entorhinal cortex in Alzheimer’s disease post-mortem brain tissue. (DOCX 24 kb)

Additional file 2: **Table S2.** Proteins identified in the pellet with increased expression in the presubiculum compared to the entorhinal cortex in Alzheimer’s disease post-mortem brain tissue. (DOCX 38 kb)

Additional file 3: **Table S3.** Proteins identified in supernatant with decreased expression in the presubiculum compared to the entorhinal cortex in Alzheimer’s disease post-mortem brain tissue. (DOCX 24 kb)

Additional file 4: **Table S4.** Proteins identified in the pellet with decreased expression in the presubiculum compared to the entorhinal cortex in Alzheimer’s disease post-mortem brain tissue. (DOCX 86 kb)

Additional file 5: **Table S5.** Webgestalt GO ontology terms showing increased expression in the presubiculum compared to the entorhinal cortex in Alzheimer’s disease post-mortem brain tissue. (DOCX 18 kb)

Additional file 6: **Table S6.** Webgestalt GO ontology terms showing decreased expression in the presubiculum compared to the entorhinal cortex in Alzheimer’s disease post-mortem brain tissue. (DOCX 18 kb)
