# Supplementary material for: The presubiculum is preserved from neurodegenerative changes in Alzheimer’s disease
Source: Acta Neuropathol Commun. 2018 Jul 20;6:62. doi: 10.1186/s40478-018-0563-8 (PMC6053705; doi:10.1186/s40478-018-0563-8)
Supplement: Supplementary file 1 — Table S1. Proteins identified in supernatant with increased expression in the presubiculum compared to the entorhinal cortex in Alzheimer’s disease post-mortem brain tissue. (DOCX 24 kb) [file 40478_2018_563_MOESM1_ESM.docx]

| **Gene ID** | **Gene name** | **Fold change** | **Gene ID** | **Gene name** | **Fold change** |
| --- | --- | --- | --- | --- | --- |
| SLC24A3 | Sodium/potassium/calcium exchanger 3 | 31.49 | DSG1 | Desmoglein-1 | 1.86 |
| LDHA | L-lactate dehydrogenase A chain | 8.29 | GNAO1 | subunit alpha | 1.86 |
| TUBA3C | Tubulin alpha-3C/D chain | 6.45 | DENND1C | DENN domain-containing protein 1C | 1.85 |
| USP35 | Ubiquitin carboxyl-terminal hydrolase 35 | 6.21 | ATP1A1 | Sodium/potassium-transporting ATPase | 1.83 |
| CNP | 2'_3'-cyclic-nucleotide 3'-phosphodiesterase | 5.81 | FCGRT | IgG receptor FcRn large subunit p51 | 1.82 |
| KPRP | Keratinocyte proline-rich protein | 5.45 | UQCRC2 | mitochondrial | 1.82 |
| SCRN1 | Secernin-1 | 5.40 | TRAP1 | Heat shock protein 75 kDa_ mitochondrial | 1.81 |
| SATB1 | DNA-binding protein SATB1 | 5.06 | SIPA1L1 | like protein 1 | 1.77 |
| CCR9 | C-C chemokine receptor type 9 | 4.94 | KRT15 | Keratin_ type I cytoskeletal 15 | 1.77 |
| NWD1 | NACHT domain- and WD repeat-containing protein 1 | 4.44 | PRSS1 | Trypsin-1 | 1.75 |
| CCDC88C | Protein Daple | 4.43 | GLUD2 | Glutamate dehydrogenase 2_ mitochondrial | 1.73 |
| GNAI2 | Guanine nucleotide-binding protein G(i) subunit alpha-2 | 3.80 | EPN3 | EPN3 protein | 1.69 |
| SMARCA2 | Probable global transcription activator SNF2L2 | 3.63 | MOG | Myelin-oligodendrocyte glycoprotein | 1.69 |
| APBB2 | Amyloid beta (A4) protein-binding_ family B_ member 2 | 3.54 | ATXN2L | Ataxin-2-like protein (Fragment) | 1.69 |
| TUBB1 | Tubulin beta-1 chain | 3.52 | ANXA6 | Annexin | 1.68 |
| TERF2 | Telomeric repeat-binding factor 2 | 3.48 | HSPA8 | Heat shock cognate 71 kDa protein | 1.66 |
| VN1R1 | Vomeronasal type-1 receptor 1 | 3.38 | CAMK2A | kinase type II subunit | 1.65 |
| PLP1 | Myelin proteolipid protein | 3.37 | ABCB4 | Phosphatidylcholine translocator ABCB4 | 1.65 |
| EPB41L3 | Band 4.1-like protein 3 | 3.16 | STXBP1 | Syntaxin-binding protein 1 | 1.64 |
| GLUD1 | Glutamate dehydrogenase 1_ mitochondrial | 3.16 | ENO2 | Gamma-enolase | 1.63 |
| INTU | Protein inturned | 3.14 | EIF4G1 | Eukaryotic translation initiation factor 4 | 1.60 |
| GDI1 | Rab GDP dissociation inhibitor alpha | 3.10 | KRT2 | Keratin_ type II cytoskeletal 2 epidermal | 1.60 |
| CDON | Cell adhesion molecule-related/down-regulated by oncogenes | 3.07 | COL6A3 | Collagen alpha-3(VI) chain | 1.60 |
| TUBA1C | Tubulin alpha-1C chain | 3.06 | CAP2 | Adenylyl cyclase-associated protein | 1.59 |
| BCS1L | Mitochondrial chaperone BCS1 | 3.03 | STX1B | Syntaxin-1B | 1.59 |
| NXPH3 | Neurexophilin-3 | 3.01 | GYPA | Glycophorin-A | 1.58 |
| BPTF | Nucleosome-remodeling factor subunit BPTF (Fragment) | 2.95 | TSPAN5 | Tetraspanin-5 | 1.56 |
| GSN | Gelsolin | 2.88 | CD9 | Tetraspanin | 1.56 |
| ATP6V1A | V-type proton ATPase catalytic subunit A | 2.84 | JUP | Junction plakoglobin | 1.56 |
| CLPB | Caseinolytic peptidase B protein homolog | 2.84 | ARHGDIA | Rho GDP-dissociation inhibitor 1 | 1.55 |
| ZSCAN5A | Zinc finger and SCAN domain-containing protein 5A | 2.83 | RYR1 | Ryanodine receptor 1 | 1.55 |
| GPR162 | G protein-coupled receptor 162_ isoform CRA_b | 2.81 | TRIM65 | Tripartite motif-containing protein 65 | 1.53 |
| CACNA1C | Voltage-dependent L-type calcium channel subunit alpha-1C | 2.73 | TTC3 | E3 ubiquitin-protein ligase TTC3 | 1.53 |
| RAC2 | Ras-related C3 botulinum toxin substrate 2 | 2.68 | RERE | Arginine-glutamic acid dipeptide repeats | 1.52 |
| NPEPPSL1 | Puromycin-sensitive aminopeptidase-like protein | 2.67 | KRT12 | Keratin_ type I cytoskeletal 12 | 1.50 |
| MADD | MAP kinase-activating death domain protein | 2.65 | CLIP2 | CAP-Gly domain-containing linker protein 2 | 1.50 |
| ZFP30 | Zinc finger protein 30 homolog (Fragment) | 2.59 |  |  |  |
| TRA@ | T-cell receptor alpha chain C region | 2.58 |  |  |  |
| RAB1A | Ras-related protein Rab-1A | 2.52 |  |  |  |
| KRT80 | Keratin_ type II cytoskeletal 80 | 2.49 |  |  |  |
| TTC27 | Tetratricopeptide repeat protein 27 | 2.48 |  |  |  |
| IPO5 | Importin-5 (Fragment) | 2.45 |  |  |  |
| Sep-07 | Septin-7 | 2.43 |  |  |  |
| ARSE | Arylsulfatase E | 2.43 |  |  |  |
| UBE3A | Ubiquitin-protein ligase E3A | 2.42 |  |  |  |
| DSC1 | Desmocollin-1 | 2.41 |  |  |  |
| SLC40A1 | Solute carrier family 40 member 1 | 2.40 |  |  |  |
| ATP6V1B2 | V-type proton ATPase subunit B_ brain isoform (Fragment) | 2.37 |  |  |  |
| DDAH1 | N(G)_N(G)-dimethylarginine dimethylaminohydrolase 1 | 2.34 |  |  |  |
| SYTL1 | Synaptotagmin-like protein 1 | 2.28 |  |  |  |
| ABCA8 | ATP-binding cassette sub-family A member 8 (Fragment) | 2.26 |  |  |  |
| USP10 | Ubiquitin carboxyl-terminal hydrolase 10 | 2.26 |  |  |  |
| TUBA4A | Tubulin alpha-4A chain | 2.26 |  |  |  |
| INPP5D | Phosphatidylinositol 3_4_5-trisphosphate 5-phosphatase 1 | 2.23 |  |  |  |
| ACTL7A | Actin-like protein 7A | 2.21 |  |  |  |
| ACTN3 | Alpha-actinin-3 | 2.18 |  |  |  |
| MBP | Myelin basic protein | 2.16 |  |  |  |
| ADGRG3 | Adhesion G protein-coupled receptor G3 | 2.16 |  |  |  |
| HSPD1 | 60 kDa heat shock protein_ mitochondrial | 2.13 |  |  |  |
| GAPDH | Glyceraldehyde-3-phosphate dehydrogenase | 2.09 |  |  |  |
| TASP1 | Threonine aspartase 1 (Fragment) | 2.08 |  |  |  |
| ACO2 | Aconitate hydratase_ mitochondrial | 2.07 |  |  |  |
| CRMP1 | Dihydropyrimidinase-related protein 1 | 2.06 |  |  |  |
| AZGP1 | Zinc-alpha-2-glycoprotein | 2.06 |  |  |  |
| TPPP | Tubulin polymerization-promoting protein | 2.06 |  |  |  |
| PLXND1 | Plexin-D1 | 2.02 |  |  |  |
| TGM4 | Protein-glutamine gamma-glutamyltransferase 4 | 2.02 |  |  |  |
| PIGT | GPI transamidase component PIG-T | 2.00 |  |  |  |

**Table S1** Proteins identified in supernatant with increased expression in the presubiculum compared to the entorhinal cortex in Alzheimer’s disease post-mortem brain tissue
