# Supplementary material for: The presubiculum is preserved from neurodegenerative changes in Alzheimer’s disease
Source: Acta Neuropathol Commun. 2018 Jul 20;6:62. doi: 10.1186/s40478-018-0563-8 (PMC6053705; doi:10.1186/s40478-018-0563-8)
Supplement: Supplementary file 2 — Table S2. Proteins identified in the pellet with increased expression in the presubiculum compared to the entorhinal cortex in Alzheimer’s disease post-mortem brain tissue. (DOCX 38 kb) [file 40478_2018_563_MOESM2_ESM.docx]

**Table S2** Proteins identified in the pellet with increased expression in the presubiculum compared to the entorhinal cortex in Alzheimer’s disease post-mortem brain tissue

| **Gene ID** | **Gene name** | **Fold change** | **Gene ID** | **Gene name** | **Fold change** |
| --- | --- | --- | --- | --- | --- |
| PRR12 | Proline-rich protein 12 | 103.48 | DCD | Dermcidin | 3.08 |
| TACC2 | Transforming acidic coiled-coil-containing | 65.52 | ITSN2 | Intersectin-2 | 3.07 |
| PRSS2 | Trypsin-2 | 38.91 | SMARCAD1 | SWI/SNF-related matrix-associated actin- | 3.06 |
| GRAMD4 | GRAM domain-containing protein 4 | 31.96 | SEMA3B | Semaphorin-3B | 3.02 |
| AGPS | Alkyldihydroxyacetonephosphate synthase_ | 31.85 | SPATA5 | Spermatogenesis-associated protein 5 | 3.01 |
| CTDSPL | CTD small phosphatase-like protein | 30.81 | CCT8 | T-complex protein 1 subunit theta | 2.99 |
| HSP90AA1 | Heat shock protein HSP 90-alpha (Fragment) | 30.02 | DPYSL4 | Dihydropyrimidinase-related protein 4 | 2.97 |
| ZFP30 | Zinc finger protein 30 homolog | 23.21 | ADAM10 | Disintegrin and metalloproteinase domain- | 2.97 |
| SLC38A10 | Putative sodium-coupled neutral amino acid | 18.05 | CNGA4 | Cyclic nucleotide-gated cation channel alpha-4 | 2.96 |
| LRRC70 | Leucine-rich repeat-containing protein 70 | 17.01 | POLQ | DNA polymerase theta | 2.93 |
| ZBTB48 | Zinc finger and BTB domain-containing protein | 14.68 | PPP4R2 | Serine/threonine-protein phosphatase 4 | 2.91 |
| OR1A1 | Olfactory receptor 1A1 | 11.31 | ATP2A1 | Sarcoplasmic/endoplasmic reticulum calcium | 2.91 |
| ZNF540 | Zinc finger protein 540 | 10.52 | VAT1 | Synaptic vesicle membrane protein VAT-1 | 2.90 |
| FMO2 | Dimethylaniline monooxygenase | 10.27 | ADAMTS15 | A disintegrin and metalloproteinase | 2.89 |
| IFT122 | Intraflagellar transport protein 122 homolog | 10.04 | ACAA1 | 3-ketoacyl-CoA thiolase_ peroxisomal | 2.86 |
| CWC27 | Peptidyl-prolyl cis-trans isomerase CWC27 | 7.52 | KRT1 | Keratin_ type II cytoskeletal 1 | 2.84 |
| BTAF1 | TATA-binding protein-associated factor 172 | 6.67 | AS3MT | Arsenite methyltransferase | 2.82 |
| KRT16 | Keratin_ type I cytoskeletal 16 | 6.58 | GRN | Granulins | 2.82 |
| ESRP2 | Epithelial splicing regulatory protein 2 | 5.87 | LMAN2 | Vesicular integral-membrane protein VIP36 | 2.82 |
| NUP188 | Nucleoporin NUP188 homolog | 5.62 | P2RX7 | P2X purinoceptor 7 | 2.81 |
| KRT9 | Keratin_ type I cytoskeletal 9 | 5.55 | SLIT3 | Slit homolog 3 protein | 2.81 |
| MEGF6 | Multiple epidermal growth factor-like domains | 5.52 | AMBP | Protein AMBP | 2.80 |
| CDON | Cell adhesion molecule-related/down-regulated | 5.45 | AIMP2 | Aminoacyl tRNA synthase complex | 2.79 |
| IQSEC2 | IQ motif and SEC7 domain-containing protein 2 | 5.10 | TBRG4 | Protein TBRG4 | 2.76 |
| C9orf172 | Uncharacterized protein C9orf172 | 5.03 | DSG1 | Desmoglein-1 | 2.74 |
| S100A9 | Protein S100-A9 | 4.99 | KRT6A | Keratin_ type II cytoskeletal 6A | 2.72 |
| C2CD2 | C2 domain-containing protein 2 | 4.94 | ERBB2 | Receptor tyrosine-protein kinase erbB-2 | 2.72 |
| CEP55 | Centrosomal protein of 55 kDa (Fragment) | 4.76 | KRT2 | Keratin_ type II cytoskeletal 2 epidermal | 2.70 |
| HRNR | Hornerin | 4.62 | MTL5 | Tesmin (Fragment) | 2.69 |
| CNTN2 | Contactin-2 | 4.25 | PANK4 | Pantothenate kinase 4 | 2.69 |
| ZC3H13 | Zinc finger CCCH domain-containing protein 13 | 4.22 | IQSEC1 | IQ motif and SEC7 domain-containing protein 1 | 2.68 |
| CEP128 | Centrosomal protein of 128 kDa | 4.10 | C19orf12 | Protein C19orf12 (Fragment) | 2.67 |
| ZNF318 | Zinc finger protein 318 | 4.07 | VPS41 | Vacuolar protein sorting-associated protein 41 | 2.64 |
| FAM65A | Protein FAM65A | 4.07 | ENO2 | Gamma-enolase | 2.63 |
| BANF1 | Barrier-to-autointegration factor | 3.96 | MPP6 | MAGUK p55 subfamily member 6 | 2.61 |
| USP32 | Ubiquitin carboxyl-terminal hydrolase 32 | 3.95 | PAM | Peptidyl-glycine alpha-amidating | 2.60 |
| NME7 | Nucleoside diphosphate kinase 7 | 3.94 | TFRC | Transferrin receptor (P90_ CD71)_ isoform CRA_c | 2.34 |
| MTERF2 | Transcription termination factor 2_ | 3.91 | E2F8 | Transcription factor E2F8 | 2.34 |
| ZNF267 | Zinc finger protein 267 | 3.79 | LMNB1 | Lamin-B1 | 2.33 |
| DDX56 | Probable ATP-dependent RNA helicase DDX56 | 3.76 | MCCC1 | Methylcrotonoyl-CoA carboxylase subunit | 2.32 |
| KRT14 | Keratin_ type I cytoskeletal 14 | 3.74 | ARHGEF40 | Rho guanine nucleotide exchange factor 40 | 2.32 |
| P4HA1 | Prolyl 4-hydroxylase subunit alpha-1 | 3.71 | BRF1 | BRF1 homolog_ subunit of RNA polymerase III | 2.31 |
| QDPR | Dihydropteridine reductase | 3.67 | DCTN2 | Dynactin subunit 2 | 2.29 |
| FXYD7 | FXYD domain-containing ion transport regulator | 3.65 | XRCC5 | X-ray repair cross-complementing protein 5 | 2.29 |
| NUP205 | Nuclear pore complex protein Nup205 | 3.59 | ALB | Serum albumin | 2.28 |
| VPS50 | Syndetin | 3.57 | VAPB | Vesicle-associated membrane protein | 2.28 |
| TSPAN5 | Tetraspanin-5 | 3.56 | CAMKV | CaM kinase-like vesicle-associated protein | 2.28 |
| MYO1B | Unconventional myosin-Ib | 3.53 | GPR61 | Probable G-protein coupled receptor 61 | 2.27 |
| ATIC | Bifunctional purine biosynthesis protein PURH | 3.51 | C3orf36 | Uncharacterized protein C3orf36 | 2.27 |
| MEGF8 | Multiple epidermal growth factor-like domains | 3.49 | TNC | Tenascin | 2.26 |
| DDX6 | Probable ATP-dependent RNA helicase DDX6 | 3.46 | VEZF1 | Vascular endothelial zinc finger 1 | 2.25 |
| AK5 | Adenylate kinase isoenzyme 5 | 3.45 | ALDOC | Fructose-bisphosphate aldolase C | 2.25 |
| APOB | Apolipoprotein B-100 | 3.43 | CADM3 | Cell adhesion molecule 3 | 2.23 |
| COL9A1 | Collagen alpha-1(IX) chain | 3.36 | ACSF2 | Acyl-CoA synthetase family member 2_ | 2.23 |
| HDAC6 | Histone deacetylase 6 | 3.30 | NRXN1 | Neurexin-1-beta | 2.21 |
| SLC16A2 | Monocarboxylate transporter 8 | 3.28 | KPNA7 | Importin subunit alpha-8 | 2.20 |
| CYP3A4 | Cytochrome P450 3A4 | 3.26 | EEF1A2 | Elongation factor 1-alpha 2 | 2.19 |
| KPNA1 | Importin subunit alpha-5 | 3.25 | ADGB | Androglobin | 2.18 |
| AGBL2 | Cytosolic carboxypeptidase 2 (Fragment) | 3.24 | ANKFY1 | Rabankyrin-5 | 2.18 |
| RETSAT | All-trans-retinol 13_14-reductase (Fragment) | 3.23 | CLTCL1 | Clathrin heavy chain 2 | 2.16 |
| TRPM3 | Transient receptor potential cation channel | 3.21 | POTEKP | Putative beta-actin-like protein 3 | 2.15 |
| FAT3 | Protocadherin Fat 3 | 3.18 | MYO5A | Unconventional myosin-Va | 2.15 |
| TRPM1 | Transient receptor potential cation channel | 3.16 | KIF1A | Kinesin-like protein KIF1A | 2.12 |
| DHX40 | Probable ATP-dependent RNA helicase DHX40 | 3.13 | RHOG | Rho-related GTP-binding protein RhoG | 2.12 |
| MADD | MAP kinase-activating death domain protein | 3.12 | ACLY | ATP-citrate synthase | 2.11 |
| HTR2C | 5-hydroxytryptamine receptor 2C | 3.12 | C20orf96 | Uncharacterized protein C20orf96 | 2.11 |
| TPT1P8 | Putative translationally control tumour | 3.09 | APC | Adenomatous polyposis coli protein | 2.10 |
|  |  |  |  |  |  |
|  |  |  |  |  |  |
|  |  |  |  |  |  |
|  |  |  |  |  |  |
|  |  |  |  |  |  |
|  |  |  |  |  |  |
|  |  |  |  |  |  |
| **Gene ID** | **Gene name** | **Fold change** | **Gene ID** | **Gene name** | **Fold change** |
| HSPH1 | Heat shock protein 105 kDa | 2.09 | LRIG1 | Leucine-rich repeats and immunoglobulin-like | 1.70 |
| ADD2 | Beta-adducin | 2.08 | EIF4G2 | Eukaryotic translation initiation factor 4 gamma | 1.70 |
| CAMK2D | Calcium/calmodulin-dependent protein kinase type II subunit delta | 2.07 | HACD3 | Very-long-chain (3R)-3-hydroxyacyl-CoA | 1.70 |
| TRIM50 | E3 ubiquitin-protein ligase TRIM50 | 2.07 | TLN2 | Talin-2 | 1.70 |
| DNAH2 | Dynein heavy chain 2_ axonemal | 2.07 | KMT2C | Histone-lysine N-methyltransferase 2C | 1.70 |
| SEPT11 | Septin-11 | 2.06 | PAIP2B | Polyadenylate-binding protein-interacting | 1.70 |
| NAT6 | N-acetyltransferase 6 | 2.06 | FOXP2 | Forkhead box protein P2 | 1.70 |
| GDE1 | Glycerophosphodiester phosphodiesterase 1 | 2.05 | CCDC150 | Coiled-coil domain-containing protein 150 | 1.69 |
| NBEAL1 | Neurobeachin-like protein 1 | 2.05 | AP2B1 | AP-2 complex subunit beta | 1.69 |
| NPR1 | Atrial natriuretic peptide receptor 1 | 2.05 | WDFY4 | WD repeat- and FYVE domain-containing | 1.69 |
| TXN | Thioredoxin | 2.04 | CC2D2A | Coiled-coil and C2 domain-containing protein | 1.69 |
| ZC3H7A | Zinc finger CCCH domain-containing protein 7A | 2.04 | ZZEF1 | Zinc finger ZZ-type and EF-hand domain- | 1.69 |
| PLCG2 | 1-phosphatidylinositol 4_5-bisphosphate | 2.04 | STK36 | Serine/threonine-protein kinase 36 | 1.69 |
| PCBP3 | Poly(rC)-binding protein 3 | 2.03 | PSG7 | Putative pregnancy-specific beta-1-glycoprotein | 1.68 |
| KRT6B | Keratin_ type II cytoskeletal 6B | 2.03 | CYTIP | Cytohesin-interacting protein | 1.68 |
| SACS | Sacsin | 2.03 | ACACA | Acetyl-CoA carboxylase 1 | 1.68 |
| FANCA | Fanconi anemia group A protein | 2.02 | NCOA6 | Nuclear receptor coactivator 6 (Fragment) | 1.68 |
| SMS | Spermine synthase (Fragment) | 2.02 | MDH2 | Malate dehydrogenase_ mitochondrial | 1.68 |
| CNOT10 | CCR4-NOT transcription complex subunit 10 | 2.01 | HSPA9 | Stress-70 protein_ mitochondrial | 1.66 |
| CXXC1 | CXXC-type zinc finger protein 1 | 2.01 | RAP1A | Ras-related protein Rap-1A (Fragment) | 1.66 |
| SYT5 | Synaptotagmin-5 | 2.01 | LRBA | Lipopolysaccharide-responsive and beige-like | 1.66 |
| AKAP13 | A-kinase anchor protein 13 | 2.00 | AAK1 | AP2-associated protein kinase 1 | 1.66 |
| GSS | Glutathione synthetase | 2.00 | PCNXL2 | Pecanex-like protein 2 | 1.66 |
| SON | Protein SON | 2.00 | TAS2R7 | Taste receptor type 2 member 7 | 1.66 |
| GRIN2B | Glutamate receptor ionotropic_ NMDA 2B | 2.00 | TLR8 | Toll-like receptor 8 | 1.65 |
| FABP4 | Fatty acid-binding protein_ adipocyte | 1.99 | PRDM1 | PR domain zinc finger protein 1 | 1.65 |
| SFXN3 | Sideroflexin-3 | 1.99 | CASD1 | CAS1 domain-containing protein 1 | 1.64 |
| TNS1 | Tensin-1 | 1.98 | VCAN | Versican core protein | 1.64 |
| MYH14 | Myosin-14 | 1.97 | STK3 | Serine/threonine-protein kinase 3 | 1.63 |
| MAP3K6 | Mitogen-activated protein kinase kinase | 1.96 | CEP295 | Centrosomal protein of 295 kDa | 1.63 |
| POTEE | POTE ankyrin domain family member E | 1.96 | DHX9 | ATP-dependent RNA helicase A | 1.63 |
| TMEM140 | Transmembrane protein 140 | 1.95 | SCAMP1 | Secretory carrier-associated membrane protein | 1.63 |
| SEPT8 | Septin-8 | 1.93 | MPI | Mannose-6-phosphate isomerase | 1.63 |
| COL20A1 | Collagen alpha-1(XX) chain | 1.93 | ETFA | Electron transfer flavoprotein subunit alpha | 1.62 |
| HADHB | Trifunctional enzyme subunit beta | 1.93 | PIGG | GPI ethanolamine phosphate transferase 2 | 1.62 |
| TRRAP | Transformation/transcription domain | 1.92 | GDPD5 | Glycerophosphodiester phosphodiesterase | 1.62 |
| FERMT2 | Fermitin family homolog 2 (Fragment) | 1.92 | NOTCH2 | Neurogenic locus notch homolog protein 2 | 1.61 |
| DOCK9 | Dedicator of cytokinesis protein 9 | 1.91 | MYDGF | Myeloid-derived growth factor | 1.61 |
| SRRM1 | Serine/arginine repetitive matrix protein 1 | 1.91 | PPP2R2A | Serine/threonine-protein phosphatase 2A | 1.60 |
| DENND4A | C-myc promoter-binding protein | 1.91 | ACCS | 1-aminocyclopropane-1-carboxylate synthase | 1.60 |
| MEGF9 | Multiple epidermal growth factor-like domains | 1.90 | VPS13A | Vacuolar protein sorting-associated protein 13A | 1.60 |
| SCD | Acyl-CoA desaturase | 1.90 | INTS8 | Integrator complex subunit 8 (Fragment) | 1.59 |
| HADH | Hydroxyacyl-coenzyme A dehydrogenase | 1.89 | PCNT | Pericentrin | 1.59 |
| FGFR4 | Fibroblast growth factor receptor 4 (Fragment) | 1.89 | AHNAK | Neuroblast differentiation-associated protein | 1.59 |
| ITGA6 | Integrin alpha-6 | 1.89 | NUP160 | Nuclear pore complex protein Nup160 | 1.59 |
| CDC42BPB | Serine/threonine-protein kinase MRCK beta | 1.88 | PFN2 | Profilin-2 | 1.59 |
| NLRP11 | NACHT_ LRR and PYD domains-containing | 1.87 | IL17RB | Interleukin-17 receptor B | 1.59 |
| LRRC28 | Leucine-rich repeat-containing protein 28 | 1.87 | MAST4 | Microtubule-associated serine/threonine- | 1.58 |
| TRIM68 | E3 ubiquitin-protein ligase TRIM68 | 1.86 | SLC25A5 | ADP/ATP translocase 2 | 1.58 |
| C1orf127 | Uncharacterized protein C1orf127 (Fragment) | 1.84 | REST | RE1-silencing transcription factor | 1.57 |
| EHD1 | EH domain-containing protein 1 | 1.84 | ACAA2 | 3-ketoacyl-CoA thiolase_ mitochondrial | 1.57 |
| PDCD6IP | Programmed cell death 6-interacting protein | 1.83 | ATP2B1 | Plasma membrane calcium-transporting ATPase | 1.56 |
| OR13C3 | Olfactory receptor 13C3 | 1.83 | NRBF2 | Nuclear receptor-binding factor 2 | 1.56 |
| H2AFY | Core histone macro-H2A.1 | 1.82 | SLC45A2 | Membrane-associated transporter protein | 1.56 |
| TNXB | Tenascin-X | 1.82 | UTF1 | Undifferentiated embryonic cell transcription | 1.56 |
| KIF5B | Kinesin-1 heavy chain | 1.80 | FTO | Alpha-ketoglutarate-dependent dioxygenase | 1.56 |
| NIPSNAP3A | Protein NipSnap homolog 3A | 1.80 | SLC3A2 | 4F2 cell-surface antigen heavy chain | 1.55 |
| PTPRN2 | Receptor-type tyrosine-protein phosphatase N2 | 1.79 | FARP2 | FERM_ RhoGEF and pleckstrin domain- | 1.55 |
| ATP5H | ATP synthase subunit d_ mitochondrial | 1.78 | HID1 | Protein HID1 | 1.55 |
| ATP2A2 | Sarcoplasmic/endoplasmic reticulum calcium | 1.78 | HAPLN2 | Hyaluronan and proteoglycan link protein 2 | 1.55 |
| CARD14 | Caspase recruitment domain-containing protein | 1.77 | TAS2R43 | Taste receptor type 2 | 1.55 |
| USP32 | Ubiquitin carboxyl-terminal hydrolase 32 | 1.77 | COX7A2L | Cytochrome c oxidase subunit 7A-related | 1.55 |
| DBX2 | Homeobox protein DBX2 | 1.77 | CACNA2D1 | Voltage-dependent calcium channel subunit | 1.54 |
| ERMP1 | Endoplasmic reticulum metallopeptidase 1 | 1.77 | PBRM1 | Protein polybromo-1 (Fragment) | 1.54 |
| GCC2 | GRIP and coiled-coil domain-containing protein | 1.76 | CD9 | CD9 antigen | 1.53 |
| PDE4DIP | Myomegalin | 1.76 | ABCC8 | ATP-binding cassette sub-family C member 8 | 1.53 |
| ARRB1 | Beta-arrestin-1 (Fragment) | 1.75 | PTPRZ1 | Receptor-type tyrosine-protein phosphatase | 1.53 |
| EEF1A1 | Elongation factor 1-alpha 1 | 1.75 | PLP1 | Myelin proteolipid protein | 1.53 |
| PABPC1L | Polyadenylate-binding protein 1-like | 1.74 | ENO3 | Beta-enolase | 1.52 |
| GSE1 | Genetic suppressor element 1 | 1.74 | SLC52A1 | Solute carrier family 52_ riboflavin transporter_ | 1.52 |
| LAIR2 | Leukocyte-associated immunoglobulin | 1.73 | SLC6A1 | Sodium- and chloride-dependent GABA | 1.51 |
| NOL6 | Nucleolar protein 6 | 1.73 | PEBP1 | Phosphatidylethanolamine-binding protein 1 | 1.51 |
| CADPS | Calcium-dependent secretion activator 1 | 1.70 | DOLPP1 | Dolichyldiphosphatase 1 (Fragment) | 1.51 |
| DDX19A | ATP-dependent RNA heliase DDX19A ( | 1.70 | GPI | Glucose-6-phosphate isomerase | 1.50 |
