# Supplementary material for: The presubiculum is preserved from neurodegenerative changes in Alzheimer’s disease
Source: Acta Neuropathol Commun. 2018 Jul 20;6:62. doi: 10.1186/s40478-018-0563-8 (PMC6053705; doi:10.1186/s40478-018-0563-8)
Supplement: Supplementary file 3 — Table S3. Proteins identified in supernatant with decreased expression in the presubiculum compared to the entorhinal cortex in Alzheimer’s disease post-mortem brain tissue. (DOCX 24 kb) [file 40478_2018_563_MOESM3_ESM.docx]

| **Gene ID** | **Gene name** | **Fold change** | **Gene ID** | **Gene name** | **Fold change** |
| --- | --- | --- | --- | --- | --- |
| SERPINB5 | Serpin B5 (Fragment) | -29.92 | PCDHGA2 | Protocadherin gamma-A2 | -1.86 |
| S100A8 | Protein S100-A8 | -14.78 | PPP2R1B | Serine/threonine-protein phosphatase 2A 65 kDa | -1.84 |
| PSAP | Prosaposin | -10.58 | WDR81 | WD repeat-containing protein 81 | -1.84 |
| PHYKPL | 5-phosphohydroxy-L-lysine phospho-lyase | -10.39 | PPP2R2A | Serine/threonine-protein phosphatase 2A 55 kDa | -1.84 |
| PANK2 | Pantothenate kinase 2_ mitochondrial | -9.94 | SYP | Synaptophysin | -1.84 |
| DOCK2 | Dedicator of cytokinesis protein 2 | -9.46 | XK | Membrane transport protein XK | -1.83 |
| ARHGAP44 | Rho GTPase-activating protein 44 | -7.14 | HSF4 | Heat shock factor protein 4 | -1.82 |
| LGALS7 | Galectin-7 | -6.70 | GLMP | Glycosylated lysosomal membrane protein | -1.81 |
| EIF2AK4 | Eukaryotic translation initiation factor 2-alpha kinase 4 | -6.12 | FABP5 | Fatty acid-binding protein_ epidermal | -1.80 |
| BET1 | BET1 homolog | -5.82 | CFAP52 | Cilia- and flagella-associated protein 52 | -1.78 |
| YTHDC2 | Probable ATP-dependent RNA helicase YTHDC2 | -5.65 | FMN1 | Formin-1 | -1.77 |
| FAM109A | Sesquipedalian-1 | -5.39 | NOMO3 | Nodal modulator 3 | -1.76 |
| MXRA8 | Matrix-remodeling-associated protein 8 | -5.04 | SLCO3A1 | Solute carrier organic anion transporter family | -1.74 |
| MYLPF | Myosin regulatory light chain 2_ skeletal muscle | -4.82 | GTF3C2 | General transcription factor 3C polypeptide 2 | -1.74 |
| S100A9 | Protein S100-A9 | -4.51 | TBC1D1 | TBC1 domain family member 1 (Fragment) | -1.71 |
| SLC6A17 | Sodium-dependent neutral amino acid transporter | -4.25 | HADH | Hydroxyacyl-coenzyme A dehydrogenase_ | -1.71 |
| SMARCA4 | Transcription activator BRG1 | -4.19 | TGM5 | Protein-glutamine gamma-glutamyltransferase 5 | -1.70 |
| GFAP | Glial fibrillary acidic protein | -3.84 | ZNF532 | Zinc finger protein 532 | -1.69 |
| TOMM40 | Mitochondrial import receptor subunit TOM40 | -3.63 | COMP | Cartilage oligomeric matrix protein | -1.69 |
| MAPT | Microtubule-associated protein tau | -3.51 | SRP72 | Signal recognition particle subunit SRP72 | -1.69 |
| ADGRE2 | Adhesion G protein-coupled receptor E2 | -3.42 | TMEM260 | Transmembrane protein 260 | -1.68 |
| CALML3 | Calmodulin-like protein 3 | -3.42 | FABP4 | Fatty acid-binding protein_ adipocyte | -1.66 |
| SNAP25 | Synaptosomal-associated protein 25 (Fragment) | -3.39 | FNDC3A | Fibronectin type-III domain-containing protein 3A | -1.65 |
| S100A11 | Protein S100-A11 | -3.30 | HBA1 | Hemoglobin subunit alpha | -1.65 |
| TANC2 | Protein TANC2 | -3.23 | HMCN2 | Hemicentin-2 | -1.64 |
| OR52M1 | Olfactory receptor 52M1 | -3.15 | YWHAH | 14-3-3 protein eta | -1.63 |
| CDK5RAP2 | CDK5 regulatory subunit-associated protein 2 | -3.14 | ASPSCR1 | Tether-containing UBX domain for GLUT4 | -1.63 |
| MTFR1 | Mitochondrial fission regulator 1 | -3.13 | GPR26 | G-protein coupled receptor 26 | -1.62 |
| PLA2G4F | Cytosolic phospholipase A2 zeta | -3.11 | ACTN1 | Alpha-actinin-1 | -1.61 |
| DNALI1 | Axonemal dynein light intermediate polypeptide 1 | -2.99 | MDH2 | Malate dehydrogenase_ mitochondrial | -1.60 |
| PSMA2 | Proteasome subunit alpha type | -2.99 | CDH17 | Cadherin-17 | -1.59 |
| COL6A6 | Collagen alpha-6(VI) chain | -2.96 | NBEAL1 | Neurobeachin-like protein 1 | -1.59 |
| DNM3 | Dynamin-3 | -2.91 | GSE1 | Genetic suppressor element 1 | -1.59 |
| CPSF3L | Integrator complex subunit 11 | -2.83 | PKM | Pyruvate kinase PKM | -1.59 |
| NRXN3 | Neurexin-3-beta | -2.82 | PLEKHG3 | Pleckstrin homology domain-containing family G | -1.59 |
| POLG2 | DNA polymerase subunit gamma-2_ mitochondrial | -2.77 | FBLN7 | Fibulin-7 | -1.58 |
| SLC45A2 | Membrane-associated transporter protein (Fragment) | -2.74 | DCP1B | mRNA-decapping enzyme 1B | -1.58 |
| CALML5 | Calmodulin-like protein 5 | -2.73 | SH3GL2 | Endophilin-A1 | -1.57 |
| LUZP4 | Leucine zipper protein 4 | -2.70 | ALDOA | Fructose-bisphosphate aldolase A (Fragment) | -1.57 |
| IPO7 | Importin-7 | -2.69 | CKMT2 | Creatine kinase S-type_ mitochondrial | -1.57 |
| PRDX3 | Thioredoxin-dependent peroxide reductase_ | -2.64 | USP25 | USP25 protein | -1.56 |
| ANKRD44 | Serine/threonine-protein phosphatase 6 regulatory | -2.64 | SLC6A15 | Sodium-dependent neutral amino acid transporter | -1.55 |
| CYP4F12 | Cytochrome P450 4F12 | -2.53 | RTN4 | Reticulon-4 | -1.54 |
| SLC12A9 | Solute carrier family 12 member 9 (Fragment) | -2.51 | OCLN | Occludin | -1.54 |
| ARL8A | ADP-ribosylation factor-like protein 8A | -2.45 | CKB | Creatine kinase B-type | -1.54 |
| SLC30A8 | Zinc transporter 8 | -2.44 | ABCD4 | ATP-binding cassette sub-family D member 4 | -1.54 |
| ATP2B1 | Plasma membrane calcium-transporting ATPase 1 | -2.42 | ROBO1 | Roundabout homolog 1 | -1.53 |
| CALM2 | Calmodulin | -2.41 | HSPA5 | 78 kDa glucose-regulated protein | -1.52 |
| VCAN | Versican core protein | -2.39 | ATP5B | ATP synthase subunit beta_ mitochondrial | -1.52 |
| MMP8 | Neutrophil collagenase (Fragment) | -2.35 | PHB | Prohibitin (Fragment) | -1.50 |
| MAP2 | Microtubule-associated protein 2 | -2.34 | FHAD1 | Forkhead-associated domain-containing protein 1 | -1.51 |
| CNOT1 | CCR4-NOT transcription complex subunit 1 | -2.32 | LRIG1 | Leucine-rich repeats and immunoglobulin-like | -1.50 |
| DNAH10 | Dynein heavy chain 10_ axonemal | -2.28 |  |  |  |
| CSTB | Cystatin-B | -2.22 |  |  |  |
| HSPA9 | Stress-70 protein_ mitochondrial | -2.22 |  |  |  |
| S100A7 | Protein S100-A7 | -2.19 |  |  |  |
| PGAM1 | Phosphoglycerate mutase 1 | -2.19 |  |  |  |
| HSPB1 | Heat shock protein beta-1 | -2.16 |  |  |  |
| IL6R | Interleukin-6 receptor subunit alpha | -2.15 |  |  |  |
| OR2A2 | Olfactory receptor 2A2 | -2.14 |  |  |  |
| UMPS | Uridine 5'-monophosphate synthase | -2.12 |  |  |  |
| OTUB1 | Ubiquitin thioesterase | -2.06 |  |  |  |
| KIAA1755 | Uncharacterized protein KIAA1755 | -2.05 |  |  |  |
| GALNTL6 | Polypeptide N-acetylgalactosaminyltransferase-like 6 | -2.00 |  |  |  |

**Table S3** Proteins identified in supernatant with decreased expression in the presubiculum compared to the entorhinal cortex in Alzheimer’s disease post-mortem brain tissue
