# Supplementary material for: The presubiculum is preserved from neurodegenerative changes in Alzheimer’s disease
Source: Acta Neuropathol Commun. 2018 Jul 20;6:62. doi: 10.1186/s40478-018-0563-8 (PMC6053705; doi:10.1186/s40478-018-0563-8)
Supplement: Supplementary file 4 — Table S4. Proteins identified in the pellet with decreased expression in the presubiculum compared to the entorhinal cortex in Alzheimer’s disease post-mortem brain tissue. (DOCX 86 kb) [file 40478_2018_563_MOESM4_ESM.docx]

**Table S4** Proteins identified in the pellet with decreased expression in the presubiculum compared to the entorhinal cortex in Alzheimer’s disease post-mortem brain tissue

| **Gene ID** | **Gene name** | **Fold change** | **Gene ID** | **Gene name** | **Fold change** | | | |
| --- | --- | --- | --- | --- | --- | --- | --- | --- |
| ANXA4 | Annexin A4 | -329.85 | RALGAPA1 | Ral GTPase-activating protein subunit alpha-1 | -12.14 | | | |
| SH3GL1 | Endophilin-A2 | -282.91 | PPL | Periplakin | -12.12 | | | |
| OR6M1 | Olfactory receptor 6M1 | -234.63 | CCDC6 | Coiled-coil domain-containing protein 6 | -11.81 | | | |
| PCSK6 | Proprotein convertase subtilisin/kexin type 6 | -141.07 | EIF2AK2 | Interferon-induced_ double-stranded RNA- | -11.72 | | | |
| WDFY3 | WD repeat and FYVE domain-containing | -120.68 | CORO1A | Coronin-1A | -11.62 | | | |
| SQSTM1 | Sequestosome-1 | -100.49 | AHCTF1 | Protein ELYS | -11.46 | | | |
| NDUFB9 | NADH dehydrogenase [ubiquinone] 1 beta | -89.73 | FBXO2 | F-box only protein 2 | -11.38 | | | |
| SLC19A3 | Thiamine transporter 2 | -72.5 | HNRNPR | Heterogeneous nuclear ribonucleoprotein R | -11.29 | | | |
| XPO1 | Exportin-1 | -58.63 | PKM | Pyruvate kinase PKM | -11.04 | | | |
| DAAM2 | Disheveled-associated activator | -55.82 | CDH18 | Cadherin-18 | -10.85 | | | |
| ACOT2 | Acyl-coenzyme A thioesterase 2 | -52.74 | MTMR10 | Myotubularin-related protein 10 | -10.69 | | | |
| MPHOSPH10 | U3 small nucleolar ribonucleoprotein protein | -51.66 | IDH3G | Isocitrate dehydrogenase [NAD] subunit | -10.64 | | | |
| WIZ | Protein Wiz | -49.97 | CHD3 | Chromodomain-helicase-DNA-binding protein | -10.62 | | | |
| UBE2M | NEDD8-conjugating enzyme Ubc12 | -49.24 | TXN2 | Thioredoxin_ mitochondrial | -10.53 | | | |
| TUBGCP6 | Gamma-tubulin complex component 6 | -47.71 | GLYAT | Glycine N-acyltransferase (Fragment) | -10.36 | | | |
| PTPN11 | Tyrosine-protein phosphatase non-receptor | -46.6 | FHL1 | Four and a half LIM domains protein 1 | -10.17 | | | |
| PITPNA | Phosphatidylinositol transfer protein alpha | -44.49 | AP3B2 | AP-3 complex subunit beta-2 | -10.04 | | | |
| GNPTAB | N-acetylglucosamine-1-phosphotransferase s | -44.25 | HNRNPH3 | Heterogeneous nuclear ribonucleoprotein H3 | -9.99 | | | |
| ENPP6 | Ectonucleotide pyrophosphatase | -38.36 | PHPT1 | 14 kDa phosphohistidine phosphatase | -9.96 | | | |
| AQP4 | Aquaporin-4 | -37.04 | GRIA1 | Glutamate receptor 1 | -9.96 | | | |
| UQCRB | Cytochrome b-c1 complex subunit 7 | -36.56 | RNF10 | RING finger protein 10 | -9.62 | | | |
| STRN3 | Striatin-3 | -35.8 | GNAI3 | Guanine nucleotide-binding protein G(k) | -9.46 | | | |
| FTL | Ferritin light chain | -33.8 | MSN | Moesin | -9.2 | | | |
| HOMEZ | Homeobox and leucine zipper protein Homez | -33.7 | PLAA | Phospholipase A-2-activating protein | -9.12 | | | |
| ZSCAN18 | Zinc finger and SCAN domain-containing | -33.07 | NANS | Sialic acid synthase (Fragment) | -8.95 | | | |
| C3 | Complement C3 | -31.4 | PRKCE | Protein kinase C epsilon type | -8.82 | | | |
| GFAP | Glial fibrillary acidic protein (Fragment) | -31.04 | OR10J4 | Olfactory receptor 10J4 | -8.8 | | | |
| GSTM2 | Glutathione S-transferase Mu 2 | -28.89 | PER1 | Period circadian protein homolog 1 | -8.64 | | | |
| Sep-10 | Septin 10_ isoform CRA_c | -26.97 | SH3GLB2 | Endophilin-B2 | -8.6 | | | |
| EIF3D | Eukaryotic translation initiation factor 3 | -25.05 | NECAB1 | N-terminal EF-hand calcium-binding protein 1 | -8.53 | | | |
| RPL4 | 60S ribosomal protein L4 | -24.36 | SNAP25 | Synaptosomal-associated protein 25 | -8.5 | | | |
| AIMP1 | Aminoacyl tRNA synthase complex | -23.91 | VPS8 | Vacuolar protein sorting-associated protein 8 | -8.42 | | | |
| RANBP1 | Ran-specific GTPase-activating protein | -22.022 | NPEPL1 | Probable aminopeptidase NPEPL1 | -8.23 | | | |
| TWF2 | Twinfilin-2 | -21.77 | ADGRB2 | Adhesion G protein-coupled receptor B2 | -8.17 | | | |
| GDAP1L1 | Ganglioside-induced differentiation protein | -21.27 | CAPN2 | Calpain-2 catalytic subunit | -8.07 | | | |
| S100B | Protein S100-B | -21.08 | GLRX5 | Glutaredoxin-related protein 5_ mitochondrial | -8.06 | | | |
| SLC9A6 | Sodium/hydrogen exchanger 6 (Fragment) | -21.08 | CPNE6 | Copine-6 | -7.99 | | | |
| STAT1 | Signal transducer and activator of transcription | -20.54 | DEK | Protein DEK | -7.81 | | | |
| ACSS3 | Acyl-CoA synthetase short-chain family | -20.5 | FGA | Fibrinogen alpha chain | -7.7 | | | |
| KIF27 | Kinesin-like protein KIF27 | -20.4 | IDH1 | Isocitrate dehydrogenase [NADP] cytoplasmic | -7.68 | | | |
| PEA15 | Astrocytic phosphoprotein PEA-15 | -20.03 | ALPK2 | Alpha-protein kinase 2 | -7.6 | | | |
| CBX8 | Chromobox protein homolog 8 | -19.7 | PSMD3 | 26S proteasome non-ATPase regulatory | -7.58 | | | |
| TOP2B | DNA topoisomerase 2 (Fragment) | -19.37 | CACNA2D3 | Voltage-dependent calcium channel subunit | -7.54 | | | |
| FGB | Fibrinogen beta chain | -19.08 | GGA1 | ADP-ribosylation factor-binding protein GGA1 | -7.52 | | | |
| KRT4 | Keratin_ type II cytoskeletal 4 | -18.93 | MAPT | Microtubule-associated protein tau | -7.48 | | | |
| KIAA1715 | Protein lunapark (Fragment) | -18.41 | MAPT | Microtubule-associated protein | -7.48 | | | |
| EHD2 | EH domain-containing protein 2 | -17.99 | RDX | Radixin | -7.27 | | | |
| PHYHIP | Phytanoyl-CoA hydroxylase-interacting protein | -17.54 | SNTA1 | Alpha-1-syntrophin | -7.22 | | | |
| GPC6 | Glypican-6 | -17.43 | SRCIN1 | SRC kinase-signaling inhibitor 1 (Fragment) | -7.09 | | | |
| SLC35E3 | Solute carrier family 35 member E3 | -17.38 | SCAMP5 | Secretory carrier-associated membrane pro | -7.06 | | | |
| APOE | Apolipoprotein E | -17.36 | EPHX1 | Epoxide hydrolase 1 | -6.91 | | | |
| RAD51D | DNA repair protein RAD51 homolog 4 | -17.32 | RAP1GAP | Rap1 GTPase-activating protein 1 | -6.9 | | | |
| UQCRFS1 | Cytochrome b-c1 complex subunit Rieske | -17.21 | MDN1 | Midasin | -6.86 | | | |
| CLTA | Clathrin light chain A | -15.47 | CYB5R3 | NADH-cytochrome b5 reductase 3 | -6.85 | | | |
| DENND5B | DENN domain-containing protein 5B | -15.39 | SPTB | Spectrin beta chain_ erythrocytic | -6.68 | | | |
| CNTNAP3 | Contactin-associated protein-like 3 | -15.3 | UTP11L | Probable U3 small nucleolar RNA-associated | -6.6 | | | |
| NADK2 | NAD kinase 2_ mitochondrial | -15.3 | DDAH2 | N(G)_N(G)-dimethylarginine | -6.59 | | | |
| ARL2 | ADP-ribosylation factor-like protein 2 | -15.1 | MAP4K4 | Mitogen-activated protein kinase 4 | -6.55 | | | |
| PSMC4 | 26S protease regulatory subunit 6B | -14.87 | KAZN | Kazrin | -6.53 | | | |
| ATL1 | Atlastin-1 | -14.84 | GNL1 | Guanine nucleotide-binding protein-like 1 | -6.51 | | | |
| Sep-02 | Septin-2 | -14.7 | EPS15L1 | Epidermal growth factor receptor substrate | | | -6.34 |  |
| GLI2 | Zinc finger protein GLI2 | -14.59 | MAPRE2 | Microtubule-associated protein RP/EB family | | | -6.34 |  |
| DLG2 | Disks large homolog 2 | -14.52 | SPTAN1 | Spectrin alpha chain_ non-erythrocytic 1 | | | -6.29 |  |
| FKBP4 | Peptidyl-prolyl cis-trans isomerase FKBP4 | -14.29 | PTK2B | Protein-tyrosine kinase 2-beta | | | -6.27 |  |
| CDS2 | Phosphatidate cytidylyltransferase 2 | -14.25 | CRKL | Crk-like protein | | | -6.24 |  |
| WDR7 | WD repeat-containing protein 7 | -13.71 | FASTKD1 | FAST kinase domain-containing protein 1 | | | -6.23 |  |
| CORO1B | Coronin-1B | -13.46 | MAP2K2 | Dual specificity mitogen-activated protein kinase | | | -6.21 |  |
| CAMK2A | Calcium/calmodulin-dependent protein kinase | -13.19 | THBS2 | Thrombospondin-2 | | | -6.06 |  |
| CAMK1D | Calcium/calmodulin-dependent protein kinase | -12.7 | LZTFL1 | Leucine zipper transcription factor-like protein 1 | | | -6.04 |  |
| LAMA4 | Laminin subunit alpha-4 | -12.64 | ERLIN1 | Erlin-1 | | | -6 |  |
| POLG | DNA polymerase subunit gamma-1 | -12.5 | CUX1 | Protein CASP | | | -5.95 |  |
| CDH23 | Cadherin-23 | -12.41 | CWH43 | PGAP2-interacting protein | | | -5.94 |  |
| HOMER1 | Homer protein homolog 1 | -12.24 | EPS15L1 | Epidermal growth factor receptor substrate 15-l | | | -6.34 |  |
|  |  |  |  |  | |  | | |
| **Gene ID** | **Gene name** | **Fold change** | **Gene ID** | **Gene name** | | **Fold change** | | |
| PNPO | Pyridoxine-5'-phosphate oxidase | -5.92 | EIF4H | Eukaryotic translation initiation | | -4.2 | | |
| PABPC1 | Polyadenylate-binding protein | -5.78 | PDE2A | cGMP-dependent 3'_5'-cyclic phosphodiesterase | | -4.19 | | |
| UTY | Histone demethylase UTY | -5.77 | DDX1 | ATP-dependent RNA helicase DDX1 | | -4.19 | | |
| NDUFA13 | NADH dehydrogenase [ubiquinone] 1 alpha | -5.75 | DYNC1LI2 | Cytoplasmic dynein 1 light intermediate chain 2 | | -4.16 | | |
| 2 | Proteasome subunit alpha type | -5.75 | ARHGAP35 | Rho GTPase-activating protein 35 | | -4.16 | | |
| PDIA3 | Protein disulfide-isomerase A3 (Fragment) | -5.65 | CRIP2 | Cysteine-rich protein 2 (Fragment) | | -4.15 | | |
| EZR | Ezrin | -5.59 | GMFB | Glia maturation factor beta | | -4.14 | | |
| ME1 | NADP-dependent malic enzyme | -5.46 | TRIM36 | E3 ubiquitin-protein ligase TRIM36 (Fragment) | | -4.13 | | |
| KIF3B | Kinesin-like protein KIF3B | -5.43 | CCP110 | Centriolar coiled-coil protein of 110 kDa | | -4.09 | | |
| PPFIA2 | Liprin-alpha-2 | -5.4 | HNRNPA3 | Heterogeneous nuclear ribonucleoprotein A3 | | -4.09 | | |
| ADAR | Double-stranded RNA-specific adenosine | -5.39 | ARFIP2 | Arfaptin-2 | | -4.081 | | |
| OTUB1 | Ubiquitin thioesterase | -5.32 | ASAP1 | Arf-GAP with SH3 domain_ ANK repeat | | -4.03 | | |
| CDH2 | Cadherin-2 | -5.27 | TJP2 | Tight junction protein ZO-2 | | -4 | | |
| HSD17B12 | Very-long-chain 3-oxoacyl-CoA reductase | -5.27 | WNK2 | Serine/threonine-protein kinase WNK2 | | -3.99 | | |
| DMXL2 | DmX-like protein 2 | -5.25 | C16orf71 | Uncharacterized protein C16orf71 | | -3.97 | | |
| TTC28 | Tetratricopeptide repeat protein 28 | -5.25 | MAPK1 | Mitogen-activated protein kinase 1 | | -3.95 | | |
| GRB2 | Growth factor receptor-bound protein 2 | -5.25 | KIAA2022 | Protein KIAA2022 | | -3.92 | | |
| SMARCA4 | Transcription activator BRG1 | -5.22 | CSDE1 | Cold shock domain-containing protein E1 | | -3.92 | | |
| TMEM30A | Cell cycle control protein 50A (Fragment) | -5.21 | LMO7 | LIM domain only protein 7 | | -3.91 | | |
| HSPA12B | Heat shock 70 kDa protein 12B | -5.2 | YWHAQ | 14-3-3 protein theta | | -3.91 | | |
| SV2A | Synaptic vesicle glycoprotein 2A | -5.18 | KCNMA1 | Calcium-activated potassium channel subunit | | -3.9 | | |
| HNRNPDL | Heterogeneous nuclear ribonucleoprotein | -5.17 | FRY | Protein furry homolog | | -3.89 | | |
| GGT5 | Gamma-glutamyltransferase 5 | -5.15 | PPFIBP1 | Liprin-beta-1 (Fragment) | | -3.89 | | |
| RALA | Ras-related protein Ral-A (Fragment) | -5.13 | NCAN | Neurocan core protein | | -3.89 | | |
| NAMPT | Nicotinamide phosphoribosyltransferase | -5.12 | PHF1 | PHD finger protein 1 | | -3.88 | | |
| SLC1A3 | Amino acid transporter | -5.1 | SH3GL2 | Endophilin-A1 | | -3.87 | | |
| PGAM1 | Phosphoglycerate mutase 1 | -5.1 | VPS35 | Vacuolar protein sorting-associated protein 35 | | -3.86 | | |
| TRPC4 | Short transient receptor potential channel 4 | -5.09 | LLGL1 | Lethal(2) giant larvae protein homolog 1 | | -3.86 | | |
| RPL18 | 60S ribosomal protein L18 (Fragment) | -5.09 | GNG12 | Guanine nucleotide-binding protein | | -3.86 | | |
| DBN1 | Drebrin | -5.08 | HDAC10 | Histone deacetylase 10 | | -3.84 | | |
| TRIM28 | Transcription intermediary factor 1-beta | -5.04 | TPM4 | Tropomyosin alpha-4 chain | | -3.83 | | |
| CBSL | Cystathionine beta-synthase-like protein | -5.02 | ERBB3 | Receptor tyrosine-protein kinase erbB-3 | | -3.83 | | |
| DNAJB6 | DnaJ homolog subfamily B member 6 | -5.02 | GSTK1 | Glutathione S-transferase kappa 1 | | -3.82 | | |
| HNRNPL | Heterogeneous nuclear ribonucleoprotein L | -4.98 | KIF5A | Kinesin heavy chain isoform 5A | | -3.8 | | |
| MYO18A | Unconventional myosin-XVIIIa | -4.94 | ANKHD1 | Ankyrin repeat and KH domain-containing | | -3.78 | | |
| PPFIBP2 | Liprin-beta-2 | -4.93 | PRKACB | cAMP-dependent protein kinase catalytic | | -3.77 | | |
| LMCD1 | LIM and cysteine-rich domains protein 1 | -4.92 | ASTE1 | Asteroid homolog 1 (Drosophila)_ isoform CRA_c | | -3.76 | | |
| LTBP4 | Latent-transforming growth factor beta | -4.91 | GNA11 | Guanine nucleotide-binding protein subunit | | -3.75 | | |
| NDUFA9 | NADH dehydrogenase [ubiquinone] 1 alpha | -4.91 | MYL12A | Myosin regulatory light chain 12A (Fragment) | | -3.74 | | |
| LRP1B | Low-density lipoprotein receptor-related | -4.9 | HNRNPCL2 | Heterogeneous nuclear ribonucleoprotein C-like | | -3.74 | | |
| NPTXR | Neuronal pentraxin receptor | -4.9 | ARHGAP1 | Rho GTPase-activating protein 1 | | -3.71 | | |
| ANKS1B | Ankyrin repeat and sterile alpha motif | -4.9 | CYFIP2 | Cytoplasmic FMR1-interacting protein 2 | | -3.69 | | |
| ACADM | Medium-chain-specific acyl-CoA | -4.81 | CCT2 | T-complex protein 1 subunit beta | | -3.67 | | |
| COL6A3 | Collagen alpha-3(VI) chain | -4.81 | PPP2R4 | Serine/threonine-protein phosphatase 2A | | -3.66 | | |
| GANAB | Neutral alpha-glucosidase AB | -4.79 | TAC4 | Tachykinin-4 | | -3.66 | | |
| PCCB | Propionyl-CoA carboxylase beta chain | -4.73 | RPL10A | 60S ribosomal protein L10a | | -3.62 | | |
| PFKFB2 | 6-phosphofructo-2-kinase | -4.7 | PLCD1 | 1-phosphatidylinositol 4_5-bisphosphate | | -3.6 | | |
| SLC6A17 | Sodium-dependent neutral amino acid | -4.6 | TPM3 | Tropomyosin alpha-3 chain | | -3.58 | | |
| TRIM2 | Tripartite motif-containing protein 2 | -4.59 | PDK2 | [Pyruvate dehydrogenase (acetyl-transferring)] | | -3.57 | | |
| BAZ1A | Bromodomain adjacent to zinc finger domain | -4.58 | CTPS2 | CTP synthase 2 | | -3.56 | | |
| NBAS | Neuroblastoma-amplified sequence | -4.56 | CAMK2B | Calcium/calmodulin-dependent protein kinase | | -3.55 | | |
| SYNM | Desmuslin_ isoform CRA_a | -4.56 | ASXL3 | Putative Polycomb group protein ASXL3 | | -3.54 | | |
| SND1 | Staphylococcal nuclease domain-containing | -4.53 | DKFZp686J | Epididymis luminal protein 189 | | -3.52 | | |
| SEPT1 | Septin-1 | -4.53 | ABCB8 | ATP-binding cassette sub-family B member 8 | | -3.52 | | |
| TSR1 | Pre-rRNA-processing protein TSR1 homolog | -4.53 | DBT | Lipoamide acyltransferase | | -3.52 | | |
| TNRC18 | Trinucleotide repeat-containing gene 18 | -4.5 | NAP1L1 | Nucleosome assembly protein 1-like 1 | | -3.51 | | |
| SLC23A1 | Solute carrier family 23 member 1 | -4.49 | DPYSL3 | Dihydropyrimidinase-related protein 3 | | -3.48 | | |
| PREPL | Prolyl endopeptidase-like | -4.48 | SEPT3 | Neuronal-specific septin-3 | | -3.47 | | |
| STIP1 | Stress-induced-phosphoprotein 1 | -4.42 | CALML3 | Calmodulin-like protein 3 | | -3.46 | | |
| PC | Pyruvate carboxylase_ mitochondrial | -4.39 | MAOB | Amine oxidase [flavin-containing] B | | -3.46 | | |
| PON2 | Paraoxonase 2_ isoform CRA_a | -4.37 | BRCA2 | Breast cancer type 2 susceptibility protein | | -3.43 | | |
| GJC2 | Gap junction gamma-2 protein | -4.37 | GRSF1 | G-rich sequence factor 1 | | -3.42 | | |
| ANXA7 | Annexin A7 | -4.36 | KPNA3 | Importin subunit alpha-4 | | -3.41 | | |
| PGRMC1 | Membrane-associated progesterone receptor | -4.36 | FMN1 | Formin-1 | | -3.4 | | |
| SPG11 | Spatacsin | -4.35 | HNRNPH1 | Heterogeneous nuclear ribonucleoprotein H | | -3.4 | | |
| PIN1 | Peptidyl-prolyl cis-trans isomerase NIMA | -4.34 | PRKAR1A | cAMP-dependent protein kinase type I-alpha | | -3.38 | | |
| CSTB | Cystatin-B | -4.34 | PFKM | ATP-dependent 6-phosphofructokinase_ muscle | | -3.37 | | |
| HDLBP | High density lipoprotein binding protein | -4.32 | NYAP1 | Neuronal tyrosine-phosphorylated | | -3.37 | | |
| GCNT4 | Beta-1_3-galactosyl-O-glycosyl-glycoprotein | -4.3 | TPT1 | Translationally-controlled tumor protein | | -3.35 | | |
| KIF5C | Kinesin heavy chain isoform 5C | -4.29 | GSTM3 | Glutathione S-transferase Mu 3 | | -3.35 | | |
| EIF5 | Eukaryotic translation initiation factor 5 | -4.27 | RUVBL1 | RuvB-like 1 | | -3.34 | | |
| GAK | Cyclin-G-associated kinase | -4.27 | CLIP1 | CAP-Gly domain-containing linker protein 1 | | -3.34 | | |
| UBA6 | Ubiquitin-like modifier-activating enzyme 6 | -4.25 | GFAP | Glial fibrillary acidic protein | | -3.32 | | |
| IQGAP2 | Ras GTPase-activating-like protein IQGAP2 | -4.24 | HARS | Histidine--tRNA ligase_ cytoplasmic | | -3.31 | | |
| ACTR2 | ARP2 actin-related protein 2 homolog (Yeast)_ | -4.2 | SAMM50 | Sorting and assembly machinery component 50 | | -3.31 | | |
| **Gene ID** | **Gene name** | **Fold change** | **Gene ID** | **Gene name** | | **Fold change** | | |
| NAP1L1 | Nucleosome assembly protein 1-like 1 | -3.51 | ATP2C1 | Calcium-transporting ATPase | | -2.86 | | |
| DPYSL3 | Dihydropyrimidinase-related protein 3 | -3.48 | NDUFS2 | NADH dehydrogenase [ubiquinone] iron-sulfur | | -2.85 | | |
| SEPT3 | Neuronal-specific septin-3 | -3.47 | ADD1 | Alpha-adducin | | -2.84 | | |
| CALML3 | Calmodulin-like protein 3 | -3.46 | YWHAE | 14-3-3 protein epsilon | | -2.82 | | |
| MAOB | Amine oxidase [flavin-containing] B | -3.46 | ARHGAP44 | Rho GTPase-activating protein 44 | | -2.79 | | |
| BRCA2 | Breast cancer type 2 susceptibility protein | -3.43 | AGAP3 | Arf-GAP with GTPase_ ANK repeat | | -2.79 | | |
| GRSF1 | G-rich sequence factor 1 | -3.42 | AKR7A2 | Aflatoxin B1 aldehyde reductase member 2 | | -2.79 | | |
| KPNA3 | Importin subunit alpha-4 | -3.41 | NRCAM | Neuronal cell adhesion molecule | | -2.78 | | |
| FMN1 | Formin-1 | -3.4 | ARHGDIA | Rho GDP-dissociation inhibitor 1 | | -2.78 | | |
| HNRNPH1 | Heterogeneous nuclear ribonucleoprotein H | -3.4 | SLC1A4 | Neutral amino acid transporter A | | -2.78 | | |
| PRKAR1A | cAMP-dependent protein kinase type I-alpha | -3.38 | DHX32 | Putative pre-mRNA-splicing factor ATP- | | -2.78 | | |
| PFKM | ATP-dependent 6-phosphofructokinase | -3.37 | CYCS | Cytochrome c (Fragment) | | -2.77 | | |
| NYAP1 | Neuronal tyrosine-phosphorylated | -3.37 | CDC37 | Hsp90 co-chaperone Cdc37 (Fragment) | | -2.74 | | |
| TPT1 | Translationally-controlled tumor protein | -3.35 | LAMB2 | Laminin subunit beta-2 | | -2.74 | | |
| GSTM3 | Glutathione S-transferase Mu 3 | -3.35 | DLG4 | Disks large homolog 4 | | -2.73 | | |
| RUVBL1 | RuvB-like 1 | -3.34 | PDIA3 | Protein disulfide-isomerase A3 | | -2.73 | | |
| CLIP1 | CAP-Gly domain-containing linker protein 1 | -3.34 | CCDC65 | Coiled-coil domain-containing protein 65 | | -2.72 | | |
| GFAP | Glial fibrillary acidic protein | -3.32 | LRRK2 | Leucine-rich repeat serine/threonine-protein | | -2.72 | | |
| HARS | Histidine--tRNA ligase_ cytoplasmic | -3.31 | PSAP | Prosaposin | | -2.7 | | |
| SAMM50 | Sorting and assembly machinery component | -3.31 | HGF | Hepatocyte growth factor (Fragment) | | -2.7 | | |
| HYOU1 | Hypoxia up-regulated protein 1 | -3.3 | MYH7B | Myosin-7B | | -2.67 | | |
| RPL30 | 60S ribosomal protein L30 | -3.3 | FGG | Fibrinogen gamma chain | | -2.67 | | |
| MS4A14 | Membrane-spanning 4-domains subfamily A | -3.3 | NPHP4 | Nephrocystin-4 | | -2.64 | | |
| SORT1 | Sortilin (Fragment) | -3.29 | ALDH1A1 | Retinal dehydrogenase 1 | | -2.64 | | |
| DES | Desmin | -3.29 | EPB41L2 | Band 4.1-like protein 2 | | -2.64 | | |
| TUBA1C | Tubulin alpha-1C chain | -3.29 | MYO9A | Unconventional myosin-IXa | | -2.63 | | |
| PAICS | Multifunctional protein ADE2 | -3.28 | OXR1 | Oxidation resistance protein 1 | | -2.63 | | |
| SARS | Serine--tRNA ligase_ cytoplasmic | -3.27 | PPIA | Peptidyl-prolyl cis-trans isomerase A | | -2.63 | | |
| CALR | Calreticulin | -3.27 | DNM2 | Dynamin-2 | | -2.63 | | |
| MAP2K1 | Dual specificity mitogen-activated protein | -3.25 | ARPC3 | Actin-related protein 2/3 complex subunit 3 | | -2.63 | | |
| OGDHL | 2-oxoglutarate dehydrogenase-like | -3.24 | PHYHIPL | Phytanoyl-CoA hydroxylase-interacting protein- | | -2.63 | | |
| AHCYL1 | Adenosylhomocysteinase 2 | -3.24 | ENO1 | Enolase 1 | | -2.62 | | |
| DLD | Dihydrolipoyl dehydrogenase_ mitochondrial | -3.23 | PSMC1 | 26S protease regulatory subunit 4 | | -2.61 | | |
| DTNB | Dystrobrevin | -3.21 | NDUFS8 | NADH dehydrogenase [ubiquinone] iron-sulfur | | -2.61 | | |
| SUGT1 | Protein SGT1 homolog | -3.21 | SEPT4 | Septin-4 | | -2.6 | | |
| HSD17B10 | 3-hydroxyacyl-CoA dehydrogenase type-2 | -3.21 | ACTC1 | Actin_ alpha cardiac muscle 1 | | -2.6 | | |
| ZBED2 | Zinc finger BED domain-containing protein 2 | -3.21 | NDUFV1 | NADH dehydrogenase (Ubiquinone) flavoprotein | | -2.59 | | |
| PSMC5 | 26S protease regulatory subunit 8 | -3.2 | COL12A1 | Collagen alpha-1(XII) chain | | -2.59 | | |
| USP5 | Ubiquitin carboxyl-terminal hydrolase 5 | -3.2 | GJA1 | Gap junction alpha-1 protein | | -2.59 | | |
| PSD | PH and SEC7 domain-containing protein 1 | -3.2 | ETFDH | Electron transfer flavoprotein-ubiquinone | | -2.59 | | |
| CADPS2 | Calcium-dependent secretion activator 2 | -3.16 | AP2A2 | AP-2 complex subunit alpha-2 | | -2.58 | | |
| YME1L1 | ATP-dependent zinc metalloprotease YME1L1 | -3.15 | PRKCA | Protein kinase C alpha type | | -2.57 | | |
| ARF4 | ADP-ribosylation factor 4 (Fragment) | -3.15 | RPS9 | 40S ribosomal protein S9 | | -2.57 | | |
| USP14 | Ubiquitin carboxyl-terminal hydrolase 14 | -3.15 | MYL6 | Myosin light polypeptide 6 | | -2.56 | | |
| NAPA | Alpha-soluble NSF attachment protein | -3.14 | RPL27 | 60S ribosomal protein L27 (Fragment) | | -2.55 | | |
| IDH3B | Isocitrate dehydrogenase [NAD] subunit | -3.13 | COPB2 | Coatomer subunit beta' | | -2.55 | | |
| RAN | GTP-binding nuclear protein Ran | -3.12 | COPB1 | Coatomer subunit beta | | -2.54 | | |
| PSMC2 | 26S protease regulatory subunit 7 | -3.11 | GNAS | Guanine nucleotide-binding protein G(s) subunit | | -2.54 | | |
| ATP6V1E1 | V-type proton ATPase subunit E 1 | -3.11 | ATP6V1C1 | V-type proton ATPase subunit C 1 | | -2.54 | | |
| RAB3C | Ras-related protein Rab-3C | -3.11 | PIP4K2A | Phosphatidylinositol 5-phosphate 4-kinase type- | | -2.53 | | |
| HSPB1 | Heat shock protein beta-1 | -3.1 | PSMC3 | 26S protease regulatory subunit 6A | | -2.53 | | |
| EXOC8 | Exocyst complex component 8 | -3.1 | PFKL | ATP-dependent 6-phosphofructokinase_ liver | | -2.53 | | |
| WDR37 | WD repeat-containing protein 37 | -3.07 | NOMO1 | Nodal modulator 1 | | -2.52 | | |
| IFT172 | Intraflagellar transport protein 172 homolog | -3.06 | MPDZ | Multiple PDZ domain protein | | -2.52 | | |
| SNX2 | Sorting nexin-2 | -3.05 | SOGA3 | Protein SOGA3 | | -2.52 | | |
| TUBB8 | Tubulin beta-8 chain | -3.05 | PRKCB | Protein kinase C beta type | | -2.52 | | |
| RAB8B | Ras-related protein Rab-8B (Fragment) | -3.04 | ABHD5 | 1-acylglycerol-3-phosphate | | -2.52 | | |
| HEPACAM | Hepatocyte cell adhesion molecule | -3.03 | MLC1 | Membrane protein MLC1 | | -2.49 | | |
| PPFIA3 | Liprin-alpha-3 | -3.03 | ASAH1 | Acid ceramidase | | -2.49 | | |
| MUT | Methylmalonyl-CoA mutase_ mitochondrial | -3.02 | SYN2 | Synapsin-2 | | -2.49 | | |
| HPCA | Neuron-specific calcium-binding protein | -3.02 | GSTM4 | Glutathione S-transferase Mu 4 | | -2.48 | | |
| PRKAR2B | cAMP-dependent protein kinase type II-beta | -3.01 | CLU | Clusterin | | -2.46 | | |
| PPP2CA | Serine/threonine-protein phosphatase 2A | -2.99 | SUCLG2 | Succinyl-CoA ligase [GDP-forming] subunit | | -2.46 | | |
| DYRK1A | Dual specificity tyrosine-phosphorylation- | -2.99 | GNAZ | Guanine nucleotide-binding protein G(z) | | -2.44 | | |
| CMAS | N-acylneuraminate cytidylyltransferase | -2.98 | MEPE | Matrix extracellular phosphoglycoprotein | | -2.44 | | |
| DDX39B | Spliceosome RNA helicase DDX39B (Fragment) | -2.96 | YWHAG | 14-3-3 protein gamma | | -2.44 | | |
| ESD | S-formylglutathione hydrolase | -2.96 | ATP6V1A | V-type proton ATPase catalytic subunit A | | -2.43 | | |
| RTN1 | Reticulon-1 | -2.96 | CAPZB | F-actin-capping protein subunit beta | | -2.41 | | |
| HNRNPU | Heterogeneous nuclear ribonucleoprotein U | -2.92 | SPG7 | Paraplegin | | -2.41 | | |
| ENDOD1 | Endonuclease domain-containing 1 protein | -2.92 | MYH3 | Myosin-3 | | -2.41 | | |
| TRIP12 | E3 ubiquitin-protein ligase TRIP12 | -2.89 | C14orf159 | UPF0317 protein C14orf159_ mitochondrial | | -2.41 | | |
| COPS4 | COP9 constitutive photomorphogenic | -2.89 | PBXIP1 | Pre-B-cell leukemia transcription factor- | | -2.41 | | |
| RDH14 | Retinol dehydrogenase 14 | -2.88 | IL6ST | Interleukin-6 receptor subunit beta | | -2.41 | | |
| RPS8 | 40S ribosomal protein S8 | -2.88 | CCBL2 | Kynurenine--oxoglutarate transaminase 3 | | -2.4 | | |
| HSP90AA1 | Heat shock protein HSP 90-alpha | -2.88 | TPPP | Tubulin polymerization-promoting protein | | -2.4 | | |
| SORD | Sorbitol dehydrogenase | -2.86 | CCT6A | T-complex protein 1 subunit zeta | | -2.39 | | |
| **Gene ID** | **Gene name** | **Fold change** | **Gene ID** | **Gene name** | | **Fold change** | | |
| PLCB1 | 1-phosphatidylinositol 4_5-bisphosphate | -2.39 | PLPP3 | Phospholipid phosphatase 3 | | -2.07 | | |
| TLR10 | Toll-like receptor 10 | -2.38 | DOCK7 | Dedicator of cytokinesis protein 7 | | -2.06 | | |
| DLG3 | Discs_ large homolog 3 | -2.38 | CEP135 | Centrosomal protein of 135 kDa | | -2.06 | | |
| CAMK2G | Calcium/calmodulin-dependent protein kinase | -2.38 | ATP1B1 | Sodium/potassium-transporting ATPase | | -2.06 | | |
| ETHE1 | Persulfide dioxygenase ETHE1_ mitochondrial | -2.37 | NCEH1 | Neutral cholesterol ester hydrolase 1 | | -2.06 | | |
| RPL11 | 60S ribosomal protein L11 | -2.37 | CFL2 | Cofilin-2 | | -2.06 | | |
| TRPV4 | Transient receptor potential cation channel | -2.37 | PGM3 | Phosphoacetylglucosamine mutase | | -2.05 | | |
| ACTR1A | Alpha-centractin | -2.36 | WDR17 | WD repeat-containing protein 17 | | -2.05 | | |
| CAPZA2 | F-actin-capping protein subunit alpha-2 | -2.36 | TMEM94 | Transmembrane protein 94 | | -2.04 | | |
| GNAQ | Guanine nucleotide-binding protein G(q) | -2.35 | GSTO1 | Glutathione S-transferase omega-1 | | -2.03 | | |
| DDX17 | Probable ATP-dependent RNA helicase DDX17 | -2.35 | UBA52 | Ubiquitin-60S ribosomal protein L40 | | -2.03 | | |
| HERC5 | E3 ISG15--protein ligase HERC5 | -2.33 | HDHD2 | Haloacid dehalogenase-like hydrolase domain- | | -2.03 | | |
| ATP1A4 | Sodium/potassium-transporting ATPase | -2.33 | VCL | Vinculin | | -2.03 | | |
| RABGAP1 | Rab GTPase-activating protein 1 | -2.32 | LYST | Lysosomal-trafficking regulator | | -2.02 | | |
| CYFIP1 | Cytoplasmic FMR1-interacting protein 1 | -2.32 | SYNE1 | Nesprin-1 | | -2.02 | | |
| ARF5 | ADP-ribosylation factor 5 | -2.31 | PPP2CB | Serine/threonine-protein phosphatase 2A | | -2 | | |
| CRNKL1 | Crooked neck-like protein 1 | -2.31 | ANXA1 | Annexin A1 | | -2 | | |
| DCLK1 | Serine/threonine-protein kinase DCLK1 | -2.31 | TMEM116 | Transmembrane protein 116 | | -2 | | |
| CKB | Creatine kinase B-type | -2.3 | GPX4 | Glutathione peroxidase | | -2 | | |
| IPO5 | Importin-5 | -2.3 | RUFY3 | Protein RUFY3 | | -2 | | |
| LGALS3 | Galectin-3 | -2.3 | STAU2 | Double-stranded RNA-binding protein Staufen | | -1.99 | | |
| PPP1R7 | Protein phosphatase 1 regulatory subunit 7 | -2.29 | MT-CO2 | Cytochrome c oxidase subunit 2 | | -1.99 | | |
| XRCC6 | X-ray repair cross-complementing protein 6 | -2.29 | PDHA1 | Pyruvate dehydrogenase E1 component | | -1.99 | | |
| AP1B1 | AP-1 complex subunit beta-1 | -2.29 | DEPTOR | DEP domain-containing mTOR-interacting | | -1.99 | | |
| VAMP3 | Vesicle-associated membrane protein 3 | -2.29 | CAND1 | Cullin-associated NEDD8-dissociated protein 1 | | -1.98 | | |
| PRMT1 | Protein arginine N-methyltransferase 1 | -2.28 | SLC9A3R1 | Na(+)/H(+) exchange regulatory cofactor NHE- | | -1.97 | | |
| ACACB | Acetyl-CoA carboxylase 2 | -2.28 | NSF | Vesicle-fusing ATPase (Fragment) | | -1.97 | | |
| ADD3 | Gamma-adducin | -2.28 | IDH2 | Isocitrate dehydrogenase [NADP] | | -1.97 | | |
| HAPLN1 | Hyaluronan and proteoglycan link protein 1 | -2.27 | OSBPL8 | Oxysterol-binding protein (Fragment) | | -1.97 | | |
| HSP90B1 | Endoplasmin | -2.27 | PDXK | Pyridoxal kinase | | -1.97 | | |
| COL6A2 | Collagen alpha-2(VI) chain | -2.26 | SEC22B | Vesicle-trafficking protein SEC22b | | -1.97 | | |
| MAPRE3 | Microtubule-associated protein RP/EB family) | -2.25 | GNG2 | Guanine nucleotide-binding protein subunit | | -1.96 | | |
| CNTRL | Centriolin | -2.25 | TAGLN3 | Transgelin | | -1.96 | | |
| ATXN10 | Ataxin-10 (Fragment) | -2.25 | HIST1H2BC | Histone H2B | | -1.96 | | |
| SH3PXD2A | SH3 and PX domain-containing protein 2A | -2.25 | TUFM | Elongation factor Tu_ mitochondrial | | -1.95 | | |
| CACYBP | Calcyclin-binding protein | -2.24 | PTPRE | Receptor-type tyrosine-protein phosphatase | | -1.95 | | |
| MACF1 | Microtubule-actin cross-linking factor | -2.23 | KLHL21 | Kelch-like protein 21 | | -1.95 | | |
| HBD | Hemoglobin subunit delta | -2.23 | BLVRB | Flavin reductase (NADPH) | | -1.95 | | |
| RPN1 | Dolichyl-diphosphooligosaccharide--protein | -2.23 | FRMD4B | FERM domain-containing protein 4B | | -1.95 | | |
| SCRN1 | Secernin-1 | -2.22 | PRG4 | Proteoglycan 4 | | -1.95 | | |
| GNAI2 | Guanine nucleotide-binding protein G(i) | -2.22 | CACUL1 | CDK2-associated and cullin domain-containing | | -1.95 | | |
| MED6 | Mediator of RNA polymerase II transcription | -2.21 | CASK | Peripheral plasma membrane protein CASK | | -1.95 | | |
| ADAMTS17 | A disintegrin and metalloproteinase with | -2.21 | PADI2 | Protein-arginine deiminase type-2 | | -1.94 | | |
| NAPB | Beta-soluble NSF attachment protein | -2.21 | SMCHD1 | Structural maintenance of chromosomes | | -1.94 | | |
| COPS2 | COP9 signalosome complex subunit 2 | -2.2 | FAM184A | Protein FAM184A | | -1.94 | | |
| TF | Serotransferrin | -2.2 | RPL9 | 60S ribosomal protein L9 (Fragment) | | -1.94 | | |
| FBF1 | Fas-binding factor 1 (Fragment) | -2.2 | TBCD | Tubulin-specific chaperone D | | -1.93 | | |
| RAB7A | Ras-related protein Rab-7a | -2.19 | PTGES3 | Prostaglandin E synthase 3 | | -1.93 | | |
| NEFH | Neurofilament heavy polypeptide | -2.19 | C4A | Complement C4-A | | -1.91 | | |
| COX7A2 | Cytochrome c oxidase subunit 7A2_ | -2.18 | SPAG9 | C-Jun-amino-terminal kinase | | -1.91 | | |
| ARF3 | ADP-ribosylation factor 3 | -2.17 | ABTB1 | Ankyrin repeat and BTB/POZ domain | | -1.91 | | |
| EHD3 | EH domain-containing protein 3 | -2.17 | PAFAH1B1 | Platelet-activating factor acetylhydrolase IB | | -1.91 | | |
| LMNB2 | Lamin B2_ isoform CRA_a | -2.17 | SPTBN4 | Spectrin beta chain_ non-erythrocytic 4 | | -1.91 | | |
| SLC8A2 | Sodium/calcium exchanger 2 | -2.16 | VIT | Vitrin | | -1.91 | | |
| TBC1D15 | TBC1 domain family member 15 | -2.16 | NIPSNAP1 | Protein NipSnap homolog 1 | | -1.9 | | |
| CTNND2 | Catenin delta-2 | -2.16 | CPNE5 | Copine-5 | | -1.9 | | |
| ATP8A1 | Phospholipid-transporting ATPase IA | -2.16 | YLPM1 | YLP motif-containing protein 1 | | -1.9 | | |
| SPTBN1 | Spectrin beta chain_ non-erythrocytic 1 | -2.16 | YWHAB | 14-3-3 protein beta/alpha | | -1.9 | | |
| AK1 | Adenylate kinase isoenzyme 1 | -2.16 | DHX34 | Probable ATP-dependent RNA helicase DHX34 | | -1.9 | | |
| ABHD14A- | Protein ABHD14A-ACY1 | -2.15 | BCAN | Brevican core protein | | -1.9 | | |
| HIST1H2BJ | Histone H2B type 1-J | -2.14 | NUMA1 | Nuclear mitotic apparatus protein 1 | | -1.89 | | |
| PCMT1 | Protein-L-isoaspartate | -2.14 | PRDX5 | Peroxiredoxin-5_ mitochondrial | | -1.89 | | |
| HIST1H4A | Histone H4 | -2.14 | CLIC4 | Chloride intracellular channel protein 4 | | -1.88 | | |
| HNRNPA2B1 | Heterogeneous nuclear ribonucleoproteins | -2.13 | TBCB | Tubulin-folding cofactor B | | -1.88 | | |
| STXBP5 | Syntaxin-binding protein 5 (Fragment) | -2.13 | LMNA | Prelamin-A/C | | -1.88 | | |
| RFX7 | DNA-binding protein RFX7 | -2.13 | BASP1 | Brain acid soluble protein 1 | | -1.88 | | |
| RPS6KA1 | Ribosomal protein S6 kinase | -2.12 | SLC12A7 | Solute carrier family 12 member 7 | | -1.88 | | |
| RAB2A | Ras-related protein Rab-2A | -2.11 | CEP290 | Centrosomal protein of 290 kDa | | -1.87 | | |
| MPP5 | MAGUK p55 subfamily member 5 | -2.1 | UTRN | Utrophin | | -1.87 | | |
| EIF3L | Eukaryotic translation initiation factor 3 | -2.1 | DNAJC13 | DnaJ homolog subfamily C member 13 | | -1.87 | | |
| CUL3 | Cullin-3 | -2.09 | PIP4K2B | Phosphatidylinositol 5-phosphate 4-kinase | | -1.87 | | |
| CBR1 | Carbonyl reductase [NADPH] 1 | -2.08 | ACTR3B | Actin-related protein 3B | | -1.86 | | |
| CD200 | OX-2 membrane glycoprotein | -2.08 | FSCN1 | Fascin | | -1.86 | | |
| KPNB1 | Importin subunit beta-1 | -2.08 | NDUFB10 | NADH dehydrogenase [ubiquinone] 1 | | -1.86 | | |
| TSN | Translin | -2.07 | NCL | Nucleolin | | -1.86 | | |
| PIKFYVE | 1-phosphatidylinositol 3-phosphate 5-kinase | -2.07 | RNH1 | Ribonuclease inhibitor | | -1.85 | | |
| **Gene ID** | **Gene name** | **Fold change** | **Gene ID** | **Gene name** | | **Fold change** | | |
| ASNA1 | ATPase ASNA1 | -1.86 | SYN1 | Synapsin-1 | | -1.65 | | |
| PCYT2 | Ethanolamine-phosphate cytidylyltransferase | -1.86 | CALM2 | Calmodulin | | -1.65 | | |
| PTK2 | Focal adhesion kinase 1 | -1.85 | IFT74 | Intraflagellar transport protein 74 homolog | | -1.65 | | |
| ROCK2 | Rho-associated protein kinase 2 | -1.85 | RAB14 | Ras-related protein Rab-14 | | -1.65 | | |
| SLC17A7 | Vesicular glutamate transporter 1 | -1.84 | ITSN1 | Intersectin-1 | | -1.64 | | |
| NEFL | Neurofilament light polypeptide | -1.84 | CFL1 | Cofilin-1 | | -1.64 | | |
| ATP5F1 | ATP synthase F(0) complex subunit B1 | -1.84 | GNB3 | Guanine nucleotide-binding protein | | -1.64 | | |
| HSPA1L | Heat shock 70 kDa protein 1-like | -1.83 | MCTP1 | Multiple C2 and transmembrane domain | | -1.64 | | |
| RAB1A | Ras-related protein Rab-1A | -1.83 | KCTD12 | BTB/POZ domain-containing protein KCTD12 | | -1.63 | | |
| MAOA | Amine oxidase [flavin-containing] A | -1.82 | CLVS1 | Clavesin-1 (Fragment) | | -1.62 | | |
| NCOR1 | Nuclear receptor corepressor 1 | -1.82 | EP400 | E1A-binding protein p400 | | -1.62 | | |
| FMNL2 | Formin-like protein 2 | -1.82 | CA2 | Carbonic anhydrase 2 | | -1.62 | | |
| TTC37 | Tetratricopeptide repeat protein 37 | -1.82 | RTN4 | Reticulon-4 | | -1.61 | | |
| MAP3K3 | Mitogen-activated protein kinase | -1.82 | PGK1 | Phosphoglycerate kinase 1 | | -1.61 | | |
| NCAM2 | Neural cell adhesion molecule 2 | -1.82 | RGS7 | Regulator of G-protein-signaling 7 | | -1.61 | | |
| ILVBL | Acetolactate synthase-like protein | -1.81 | DUSP3 | Dual specificity protein phosphatase 3 | | -1.61 | | |
| EEF1D | Elongation factor 1-delta | -1.81 | STRAP | Serine-threonine kinase receptor-associated | | -1.61 | | |
| ACAD8 | Isobutyryl-CoA dehydrogenase_ mitochondrial | -1.81 | TTLL6 | Tubulin polyglutamylase TTLL6 | | -1.6 | | |
| KBTBD11 | Kelch repeat and BTB domain-containing | -1.81 | ECHS1 | Enoyl-CoA hydratase_ mitochondrial | | -1.6 | | |
| CANX | Calnexin | -1.81 | G6PD | Glucose-6-phosphate 1-dehydrogenase | | -1.59 | | |
| GPD2 | Glycerol-3-phosphate dehydrogenase | -1.8 | SYP | Synaptophysin | | -1.58 | | |
| P4HB | Protein disulfide-isomerase | -1.8 | PAFAH1B3 | Platelet-activating factor acetylhydrolase IB | | -1.58 | | |
| OXCT1 | Succinyl-CoA:3-ketoacid coenzyme A | -1.8 | TCP1 | T-complex protein 1 subunit alpha | | -1.58 | | |
| ERP44 | Endoplasmic reticulum resident protein 44 | -1.8 | ATP6V1H | V-type proton ATPase subunit H | | -1.58 | | |
| VAMP2 | Vesicle-associated membrane protein 2 | -1.8 | IDH3A | Isocitrate dehydrogenase [NAD] subunit | | -1.57 | | |
| CHCHD3 | MICOS complex subunit | -1.79 | GLUL | Glutamine synthetase | | -1.57 | | |
| UCHL1 | Ubiquitin carboxyl-terminal hydrolase isozyme | -1.79 | ME2 | NAD-dependent malic enzyme_ mitochondrial | | -1.57 | | |
| RPS2 | 40S ribosomal protein S2 | -1.78 | SYNGAP1 | Ras/Rap GTPase-activating protein SynGAP | | -1.56 | | |
| LRRK1 | Leucine-rich repeat serine/threonine-protein | -1.78 | SYNJ1 | Synaptojanin-1 | | -1.56 | | |
| TOMM70A | Mitochondrial import receptor subunit TOM70 | -1.78 | ANK2 | Ankyrin-2 | | -1.56 | | |
| NAP1L4 | Nucleosome assembly protein 1-like 4 | -1.77 | AKR1A1 | Alcohol dehydrogenase [NADP(+)] | | -1.56 | | |
| MAPK3 | Mitogen-activated protein kinase 3 | -1.77 | SLC25A6 | ADP/ATP translocase 3 | | -1.55 | | |
| LAMC1 | Laminin subunit gamma-1 | -1.77 | RAP1GDS1 | Rap1 GTPase-GDP dissociation stimulator 1 | | -1.55 | | |
| GPR98 | G-protein coupled receptor 98 | -1.77 | TUBB4A | Tubulin beta-4A chain | | -1.54 | | |
| METTL7A | Methyltransferase-like protein 7A (Fragment) | -1.76 | NDUFS3 | NADH dehydrogenase [ubiquinone] iron-sulfur | | -1.54 | | |
| DAPK1 | Death-associated protein kinase 1 | -1.76 | SMC4 | Structural maintenance of chromosomes | | -1.54 | | |
| UBA1 | Ubiquitin-like modifier-activating enzyme 1 | -1.76 | STRC | Stereocilin | | -1.54 | | |
| PRKRA | Interferon-inducible double-stranded RNA | -1.76 | SFXN1 | Sideroflexin-1 | | -1.53 | | |
| FHAD1 | Forkhead-associated domain-containing | -1.74 | DNM1 | Dynamin-1 | | -1.53 | | |
| SKP1 | S-phase kinase-associated protein 1 | -1.74 | SDHA | Succinate dehydrogenase [ubiquinone] | | -1.53 | | |
| PRDX6 | Peroxiredoxin-6 | -1.74 | ACOX3 | Peroxisomal acyl-coenzyme A oxidase 3 | | -1.53 | | |
| HMGCR | 3-hydroxy-3-methylglutaryl-coenzyme A | -1.74 | UGDH | UDP-glucose 6-dehydrogenase (Fragment) | | -1.52 | | |
| GSN | Gelsolin | -1.74 | PITPNB | Phosphatidylinositol transfer protein beta i | | -1.52 | | |
| MTHFD1 | C-1-tetrahydrofolate synthase_ cytoplasmic | -1.74 | SLC25A4 | ADP/ATP translocase 1 | | -1.52 | | |
| NCALD | Neurocalcin-delta | -1.73 | GNB1 | Guanine nucleotide-binding protein | | -1.52 | | |
| PRDX2 | Peroxiredoxin-2 | -1.73 | APOO | MICOS complex subunit MIC26 | | -1.52 | | |
| LRRC37B | Leucine-rich repeat-containing protein 37B | -1.72 | H2AFV | Histone H2A | | -1.52 | | |
| EEF1G | Elongation factor 1-gamma | -1.72 | RPL18A | 60S ribosomal protein L18a | | -1.52 | | |
| CCT3 | T-complex protein 1 subunit gamma | -1.72 | DARS | Aspartate--tRNA ligase_ cytoplasmic | | -1.52 | | |
| PSAT1 | Phosphoserine aminotransferase | -1.72 | STX1A | Syntaxin-1A | | -1.52 | | |
| MSRA | Mitochondrial peptide methionine sulfoxide | -1.72 | F8 | Coagulation factor VIII | | -1.51 | | |
| CCNE2 | CCNE2 protein | -1.72 | FOLH1 | Glutamate carboxypeptidase 2 | | -1.51 | | |
| INA | Alpha-internexin | -1.72 | CCT7 | T-complex protein 1 subunit eta | | -1.51 | | |
| EIF4A2 | Eukaryotic initiation factor 4A-II | -1.71 | PCBP1 | Poly(rC)-binding protein 1 | | -1.51 | | |
| LAP3 | Cytosol aminopeptidase (Fragment) | -1.71 | ARF6 | ADP-ribosylation factor 6 | | -1.51 | | |
| CDK5 | Cyclin-dependent-like kinase 5 | -1.71 | PPP2R1A | Serine/threonine-protein phosphatase 2A 65 | | -1.5 | | |
| TSSC1 | Protein TSSC1 | -1.71 |  |  | |  | | |
| CDC42BPA | Serine/threonine-protein kinase MRCK alpha | -1.71 |  |  | |  | | |
| HBB | Hemoglobin subunit beta | -1.7 |  |  | |  | | |
| VDAC1 | Voltage-dependent anion-selective channel | -1.7 |  |  | |  | | |
| PHB2 | Prohibitin-2 | -1.7 |  |  | |  | | |
| CD81 | Tetraspanin | -1.7 |  |  | |  | | |
| TUBA8 | Tubulin alpha-8 chain (Fragment) | -1.7 |  |  | |  | | |
| BRMS1 | Breast cancer metastasis-suppressor 1 | -1.7 |  |  | |  | | |
| PYGM | Glycogen phosphorylase_ muscle form | -1.69 |  |  | |  | | |
| LTA4H | Leukotriene A-4 hydrolase | -1.69 |  |  | |  | | |
| ANXA6 | Annexin A6 | -1.68 |  |  | |  | | |
| RPL7A | 60S ribosomal protein L7a | -1.68 |  |  | |  | | |
| USP9X | Probable ubiquitin carboxyl-terminal | -1.68 |  |  | |  | | |
| PFKP | ATP-dependent 6-phosphofructokinase | -1.68 |  |  | |  | | |
| GNAI1 | Guanine nucleotide-binding protein G(i) | -1.68 |  |  | |  | | |
| PYGB | Glycogen phosphorylase_ brain form | -1.67 |  |  | |  | | |
| ACTN1 | Alpha-actinin-1 | -1.66 |  |  | |  | | |
| USP44 | Ubiquitin carboxyl-terminal hydrolase 44 | -1.66 |  |  | |  | | |
| CSRP1 | Cysteine and glycine-rich protein 1 | -1.65 |  |  | |  | | |
| HSP90AB1 | Heat shock protein HSP 90-beta | -1.65 |  |  | |  | | |
