# Supplementary material for: The presubiculum is preserved from neurodegenerative changes in Alzheimer’s disease
Source: Acta Neuropathol Commun. 2018 Jul 20;6:62. doi: 10.1186/s40478-018-0563-8 (PMC6053705; doi:10.1186/s40478-018-0563-8)
Supplement: Supplementary file 5 — Table S5. Webgestalt GO ontology terms showing increased expression in the presubiculum compared to the entorhinal cortex in Alzheimer’s disease post-mortem brain tissue. (DOCX 18 kb) [file 40478_2018_563_MOESM5_ESM.docx]

**Table S5** Webgestalt GO ontology terms showing increased expression in the presubiculum compared to the entorhinal cortex in Alzheimer’s disease post-mortem brain tissue

| *Soluble fraction* | | | | | | | |
| --- | --- | --- | --- | --- | --- | --- | --- |
|  |  |  |  |  |  |  |  |
| **Molecular function** | | | | | | | |
| **GO ID** | **GO term** |  | |  |  |  |  |
| GO:0004111 | creatine kinase activity |  | |  |  |  |  |
| GO:0004859 | phospholipase inhibitor activity |  | |  |  |  |  |
| GO:0016775 | phosphotransferase activity, nitrogenous group as acceptor |  | |  |  |  |  |
| GO:0055102 | lipase inhibitor activity |  | |  |  |  |  |
| GO:0004857 | enzyme inhibitor activity |  | |  |  |  |  |
| GO:0046915 | transition metal ion transmembrane transporter activity |  | |  |  |  |  |
| GO:0005546 | phosphatidylinositol-4,5-bisphosphate binding |  | |  |  |  |  |
| GO:0005544 | calcium-dependent phospholipid binding |  | |  |  |  |  |
| GO:0017016 | Ras GTPase binding |  | |  |  |  |  |
| GO:0031267 | small GTPase binding |  | |  |  |  |  |
|  |  |  | |  |  |  |  |
| *Insoluble fraction* |  |  | |  |  |  |  |
|  |  |  | |  |  |  |  |
| **Biological process** |  | **Molecular function** |  | |  | **Cellular component** |  |
| **GO ID** | **GO term** | **GO ID** | **GO term** | |  | **GO ID** | **GO term** |
| GO:0044281 | small molecule metabolic process | GO:0043168 | anion binding | |  | GO:0005856 | cytoskeleton |
| GO:0009152 | purine ribonucleotide biosynthetic process | GO:0005198 | structural molecule activity | |  | GO:0044430 | cytoskeletal part |
| GO:0022607 | cellular component assembly | GO:0036094 | small molecule binding | |  | GO:0044444 | cytoplasmic part |
| GO:0034329 | cell junction assembly | GO:0036094 | small molecule binding | |  | GO:0005737 | cytoplasm |
| GO:0043269 | regulation of ion transport | GO:0000166 | nucleotide binding | |  | GO:0033267 | axon part |
| GO:0010035 | response to inorganic substance | GO:1901265 | nucleoside phosphate binding | |  | GO:0030424 | axon |
| GO:0009260 | ribonucleotide biosynthetic process | GO:0032549 | ribonucleoside binding | |  | GO:0005882 | intermediate filament |
| GO:0046390 | ribose phosphate biosynthetic process | GO:0032550 | purine ribonucleoside binding | |  | GO:0044463 | cell projection part |
| GO:0044085 | cellular component biogenesis | GO:0016462 | pyrophosphatase activity | |  | GO:0045111 | intermediate filament cytoskeleton |
| GO:0034330 | cell junction organization | GO:0001882 | nucleoside binding | |  | GO:0043005 | neuron projection |
