# Supplementary material for: The presubiculum is preserved from neurodegenerative changes in Alzheimer’s disease
Source: Acta Neuropathol Commun. 2018 Jul 20;6:62. doi: 10.1186/s40478-018-0563-8 (PMC6053705; doi:10.1186/s40478-018-0563-8)
Supplement: Supplementary file 6 — Table S6. Webgestalt GO ontology terms showing decreased expression in the presubiculum compared to the entorhinal cortex in Alzheimer’s disease post-mortem brain tissue. (DOCX 18 kb) [file 40478_2018_563_MOESM6_ESM.docx]

**Table S6** Webgestalt GO ontology terms showing decreased expression in the presubiculum compared to the entorhinal cortex in Alzheimer’s disease post-mortem brain tissue

| *Soluble fraction* | | | | | |
| --- | --- | --- | --- | --- | --- |
|  |  |  |  |  |  |
| **Biological process** |  | **Molecular function** |  | **Cellular component** |  |
| **GO ID** | **GO term** | **GO ID** | **GO term** | **GO ID** | **GO term** |
| GO:0035637 | multicellular organismal signaling | GO:0005200 | structural constituent of cytoskeleton | GO:0033267 | axon part |
| GO:0019226 | transmission of nerve impulse | GO:0005198 | structural molecule activity | GO:0043005 | neuron projection |
| GO:0006836 | neurotransmitter transport | GO:0050786 | RAGE receptor binding | GO:0044463 | cell projection part |
| GO:0007268 | synaptic transmission | GO:0005515 | protein binding | GO:0045202 | synapse |
| GO:0007267 | cell-cell signaling | GO:0005509 | calcium ion binding | GO:0005829 | cytosol |
| GO:0048812 | neuron projection morphogenesis | GO:0008022 | protein C-terminus binding | GO:0005882 | intermediate filament |
| GO:0045103 | intermediate filament-based process | GO:0042287 | MHC protein binding | GO:0030424 | axon |
| GO:0050877 | neurological system process | GO:0005504 | fatty acid binding | GO:0033269 | internode region of axon |
| GO:0045104 | intermediate filament cytoskeleton organization | GO:0019829 | cation-transporting ATPase activity | GO:0045111 | intermediate filament cytoskeleton |
| GO:0032990 | cell part morphogenesis |  |  | GO:0005856 | cytoskeleton |
|  |  |  |  |  |  |
| *Insoluble fraction* |  |  |  |  |  |
|  |  |  |  |  |  |
| **Biological process** |  | **Molecular function** |  | **Cellular component** |  |
| **GO ID** | **GO term** | **GO ID** | **GO term** | **GO ID** | **GO term** |
| GO:0048666 | neuron development | GO:0005515 | protein binding | GO:0005737 | cytoplasm |
| GO:0071842 | cellular component organization at cellular level | GO:0043168 | anion binding | GO:0044444 | cytoplasmic part |
| GO:0071841 | cellular component organization or biogenesis at cellular level | GO:0000166 | nucleotide binding | GO:0005622 | intracellular |
| GO:0071840 | cellular component organization or biogenesis | GO:1901265 | nucleoside phosphate binding | GO:0044424 | intracellular part |
| GO:0016043 | cellular component organization | GO:0032553 | ribonucleotide binding | GO:0005829 | cytosol |
| GO:0051649 | establishment of localization in cell | GO:0032555 | purine ribonucleotide binding | GO:0005856 | cytoskeleton |
| GO:0030030 | cell projection organization | GO:0017076 | purine nucleotide binding | GO:0044430 | cytoskeletal part |
| GO:0009056 | catabolic process | GO:0036094 | small molecule binding | GO:0032991 | macromolecular complex |
| GO:0022008 | neurogenesis | GO:0001883 | purine nucleoside binding | GO:0043234 | protein complex |
| GO:0051641 | cellular localization | GO:0032549 | ribonucleoside binding | GO:0044422 | organelle part |
